# A case-based course teaching integrative taxonomy delimits three new ant species in the *Pheidole sexspinosa* complex (Hymenoptera, Formicidae) from Vanuatu

**DOI:** 10.7717/peerj.21333

**Published:** 2026-06-18

**Authors:** Kyle W. Gray, Sarah von Adelmannsfelden, Abdullah Ahmed, Jonathan Brandstetter, Tabea Gularte Alvarez, Max P. Härtel, Zoya Mahmood, Daniela Mera-Rodríguez, Christian Rabeling

**Affiliations:** 1 KomBioTa—Center for Biodiversity and Integrative Taxonomy Research, State Museum of Natural History Stuttgart & University of Hohenheim, Stuttgart, Baden-Württemberg, Germany; 2 Department of Integrative Taxonomy and Biodiversity of Insects, University of Hohenheim, Stuttgart, Baden-Württemberg, Germany

**Keywords:** Biodiversity, Taxonomy, Morphometrics, Biogeography, Endemism, Pheidole, Vanuatu, UCE

## Abstract

There is a dwindling number of trained taxonomists across the globe, which is part of the “taxonomic impediment” to biodiversity discovery and conservation. Therefore, there is a need to train students in taxonomy and simultaneously make the taxonomic process more robust, thus reducing the misidentification of species that leads to flawed conclusions about biogeographic, ecological, and evolutionary processes. This is where an interdisciplinary approach to taxonomy, *i.e*., “integrative taxonomy,” is useful because it combines traditional comparative morphology approaches with molecular phylogenetics as well as ecological and biogeographic information to resolve species boundaries more accurately. Here, we present a study that involved teaching integrative taxonomic methods in higher education utilizing a collaborative and case-based learning approach. Specifically, we provided students with a real taxonomic case study that focused on ants in the *Pheidole sexspinosa* complex from the Vanuatuan archipelago in the southwestern Pacific region. Based on a population-level phylogeny using ultraconserved elements (UCEs) as well as quantitative morphometrics and biogeography, we delimited three new species in the *Pheidole sexspinosa* complex from Vanuatu that we describe here as *P. epaoensis* **sp. nov.**, *P. nivanuatu* **sp. nov.**, and *P. tanakarensis* **sp. nov.** We also inferred a Cytochrome C Oxidase Subunit I (COI) phylogeny that includes additional populations of the *P. sexspinosa* complex from outside of Vanuatu, which supports the hypothesis that the Vanuatuan species diversified *in situ* within the archipelago. In addition, we discuss the need for an integrative taxonomic evaluation of the broader *Pheidole sexspinosa* complex as well as the utility of a collaborative and case-based learning approach in higher education to teach integrative taxonomy and biodiversity science.

## Introduction

The accelerating loss of species and ecosystem function worldwide has intensified the global biodiversity crisis, necessitating urgent and coordinated conservation action (Barnosky et al., 2011; Ceballos, Ehrlich & Dirzo, 2017). To better inform conservation priorities, there is a need for taxonomic efforts to describe and catalogue Earth’s vanishing biodiversity as well as to better understand its evolutionary history (Daly et al., 2012). However, less than half of the globe’s multicellular eukaryotic biodiversity has been formally described with previous estimates suggesting possibly even as little as 10% of all estimated species (González-Oreja, 2008; Mora et al., 2011). This problem is exacerbated by the dwindling number of trained taxonomists as well as resources for taxonomy, which is part of the problem behind the “taxonomic impediment” to biodiversity discovery and conservation (Engel et al., 2021). Therefore, there is a need to accelerate the taxonomic process and simultaneously make it more robust to reduce the misidentification of species that leads to flawed conclusions about biogeographic, ecological, and evolutionary processes. This is where an interdisciplinary approach to taxonomy, *i.e*., “integrative taxonomy,” is useful because it combines multiple methods such as traditional comparative morphology, molecular phylogenetics, as well as ecological and biogeographic information to resolve species boundaries more accurately (Dayrat, 2005; Schlick-Steiner et al., 2010).

**Figure 1:**
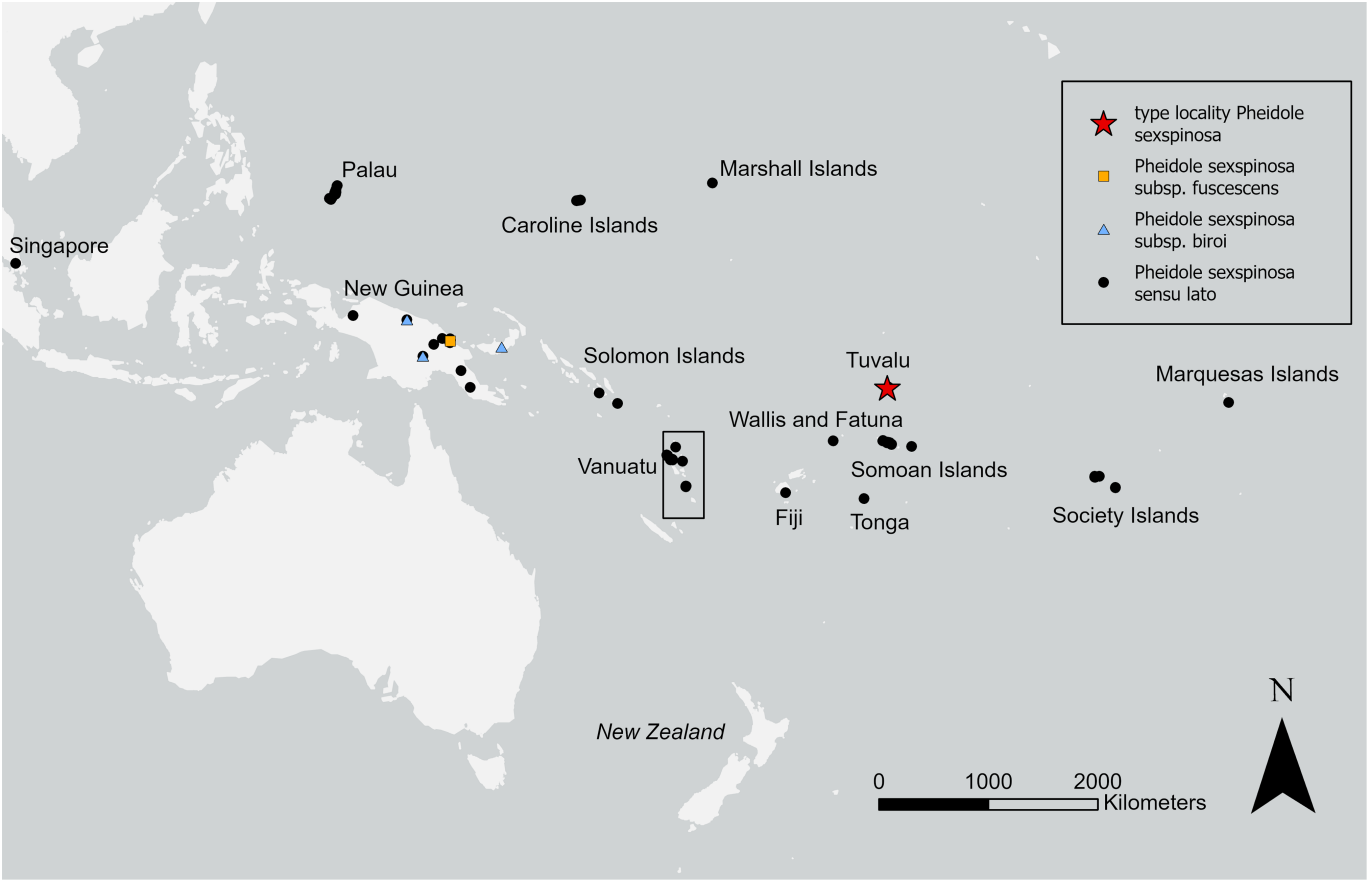
Map of the South Pacific region including the Vanuatuan archipelago (rectangle) with labeled regions and localities that the *Pheidole sexspinosa* complex has been reported to occur. Black dots represent *Pheidole sexspinosa sensu lato*, the orange square represents *P. sexspinosa* subsp. *fuscescens*, blue triangles represent *P. sexspinosa* subsp. *biroi*, and the red star represents the type locality of *Pheidole sexspinosa* *sensu* *stricto* on Tuvalu.

To help address the “taxonomic impediment” to biodiversity discovery and conservation, there has been a renewed interest in teaching taxonomy to students with an emphasis on going beyond the classic “one-on-one” mentorship model towards a more collaborative model involving an entire classroom of students (Williams et al., 2025). Here, we build upon this framework by presenting a study that involved teaching an integrative approach to taxonomy in higher education utilizing collaborative and case-based learning (Allchin, 2013; Chodkowski et al., 2022), which uses real problems for hands-on learning and focuses on teaching students with little to no previous taxonomic experience. For our taxonomic case study, we focused on ants (Hymenoptera, Formicidae) from the Vanuatuan archipelago in the southwestern Pacific region (Fig. 1). Ants are among the most ecologically dominant insects worldwide and are widely used as indicators of biodiversity and ecosystem functioning (Agosti et al., 2000; Hölldobler & Wilson, 1990). Despite this importance, the ant fauna of Vanuatu has remained severely understudied, even though Vanuatu has been predicted to be a missing center of ant biodiversity and lies at a major biogeographic crossroad between several island systems (Kass et al., 2022). Recent targeted sampling and phylogenomic analyses have begun to fill this gap, revealing that Vanuatu is indeed a significant center of ant biodiversity in the southwestern Pacific (Gray, 2023). These efforts have also uncovered numerous ant species likely representing undescribed endemics that need formal taxonomic evaluation. Specifically, we focused here on the Vanuatuan populations of the little-studied *Pheidole sexspinosa* species complex (*sensu* Economo et al., 2015; Sarnat et al., 2017) (Fig. 1), a group whose morphological and geographic variation provides a compelling case for integrative taxonomic evaluation.

*Pheidole* Westwood, 1839 (Hymenoptera, Formicidae, Myrmicinae, Attini) is a hyperdiverse and cosmopolitan ant genus with species that possess discrete minor and major worker castes that perform specialized tasks (Wilson, 1984, 2003). Species of the *Pheidole sexspinosa* complex are distributed throughout the Pacific region (Fig. 1) and currently include one nominal but extremely variable species, *P. sexspinosa* Mayr, 1870, as well as two subspecies, *P. s. biroi* Emery, 1900 and *P. s. fuscescens* Emery, 1900. *Pheidole sexspinosa* as currently recognized has a vast range spanning from Singapore in continental southeastern Asia to the Marquesas Islands in French Polynesia, whereas *P. s. biroi* and *P. s. fuscescens* occur in New Guinea (Fig. 1). Historically, the primary distinction between *P. sexspinosa* and its subspecies was coloration with *P. sexspinosa* being darker and lacking a white to yellow first gastric tergite as observed in *P. s. biroi* and *P. s. fuscescens* (Mayr, 1870; Emery, 1900). *Pheidole s. biroi* and *P. s. fuscescens* were differentiated based primarily on size with *P. s. biroi* being smaller than *P. s. fuscescens* (Emery, 1900). Since these initial taxonomic studies of the *P. sexspinosa* complex, however, most newly discovered populations of the complex have been classified as *P. sexspinosa* without further taxonomic evaluation of potential new species. The primary exception focused on recently discovered Singaporean populations (Wang, Yamada & Eguchi, 2018), which noted multiple distinct morphological traits of local populations such as the degree of facial and mesosomal sculpturing, but the authors classified these populations as *P. sexspinosa* based on COI barcode similarity to populations referred to as *P. sexspinosa* from Palau (*sensu* Economo et al., 2015).

**Figure 2:**
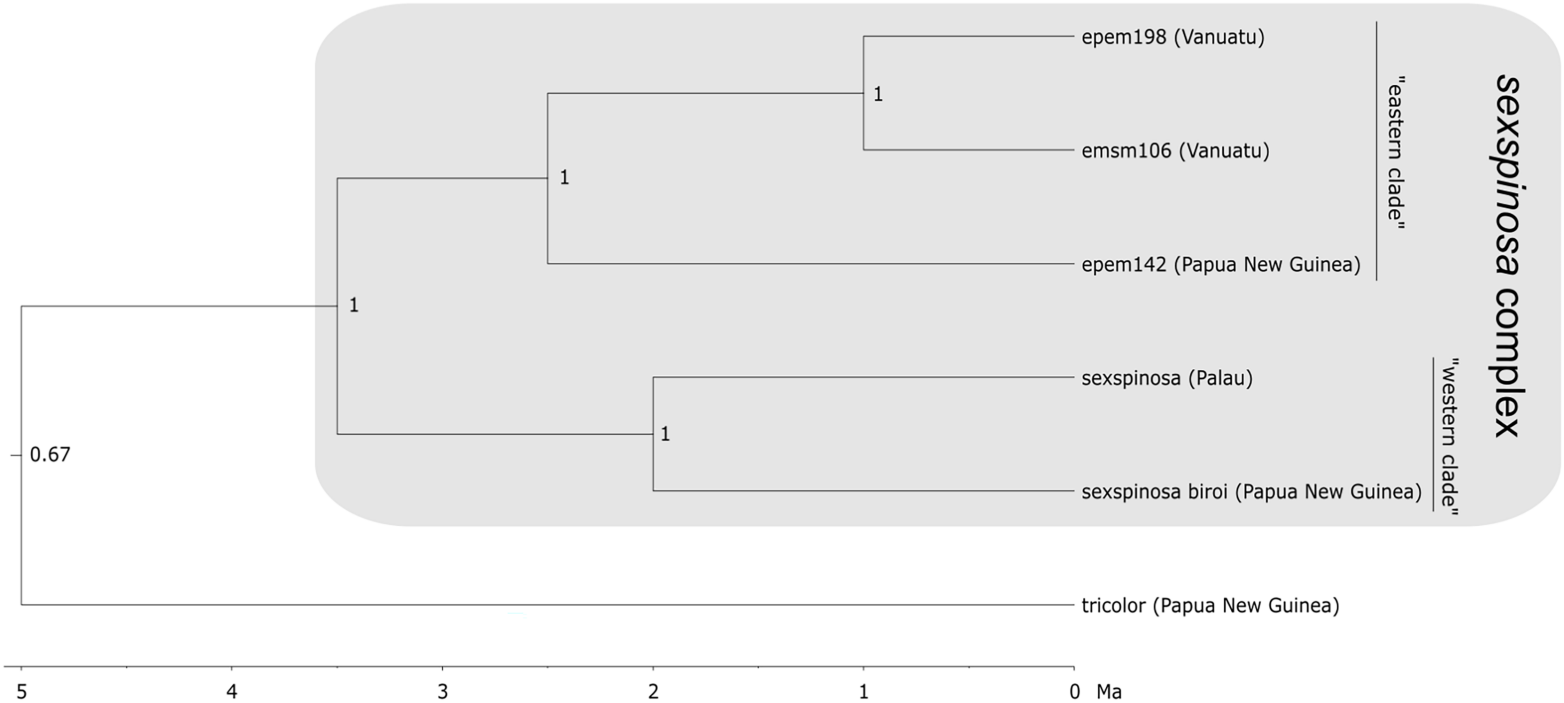
Phylogeny of the *Pheidole sexspinosa* complex and its sister species *P. tricolor* redrawn from Economo et al. (2015) with node support values shown. Tip labels include species names or morphospecies names (epem198 = CR111116-19-01; emsm106 = CR111116-43) from Economo et al. (2015) and the country of origin for each sample. Time scale is in millions of years (Ma).

Based on a phylogenetic study of Old World *Pheidole* species (Economo et al., 2015), there appear to be two subclades within the *P. sexspinosa* species complex reflecting at least two independently evolving groups and the presence of multiple cryptic and undescribed species (Fig. 2). One subclade, which we refer to as the “western clade,” includes what has been considered as *P. sexspinosa* from Palau as well as *P. s. biroi* from New Guinea (*P. s. fuscescens* was not included in the referred phylogenetic study). The other subclade, which we refer to as the “eastern clade,” consists of Vanuatuan populations of the complex (“epem198” & “emsm106”) that were inferred as sister to an undescribed lineage from Papua New Guinea (“epem142”) (Fig. 2). The Vanuatuan samples “epem198” and “emsm106” represent two morphospecies belonging to the *P. sexspinosa* complex that were collected at Mt. Tanakar on Espiritu Santo (collection codes CR111116-19-01, CR111116-43). Based on this first analysis, it appears that the Vanuatuan members of the *P. sexspinosa* complex are phylogenetically distinct from what is currently considered as *P. sexspinosa* and likely have an independent evolutionary history from other described populations of the complex. However, it remains untested whether the Vanuatuan populations of the *P. sexspinosa* complex consists of one or multiple distinct species.

Here, through case-based learning and instruction of integrative taxonomy with a small cohort of university students, we combined phylogenomics with quantitative morphometrics and biogeography to delimit species of the Vanuatuan *Pheidole sexspinosa* complex. First, we inferred a population-level phylogeny of the Vanuatuan *P. sexspinosa* complex using ultraconserved elements (“UCEs”) as molecular markers, which are thousands of conserved sequences scattered across the genome (Faircloth et al., 2012). Second, we applied comparative and quantitative morphological analyses to discern if there is the presence of distinct morphological clusters that correspond to monophyletic groups in the UCE phylogeny. Third, we tested for gene flow between and within the phylogenetically and morphologically distinct forms. Fourth, we combined the Vanuatuan UCE dataset with COI barcodes from additional specimens from across the range of the *P. sexspinosa* complex to contextualize the phylogenetic position of the Vanuatuan populations. Finally, we discuss the need for an integrative taxonomic evaluation of the broader *P. sexspinosa* complex as well as the utility of using a collaborative and case-based learning approach in higher education to teach integrative taxonomy and biodiversity science.

## Materials and Methods

### Course design for case-based learning of integrative taxonomy

The case-based learning course of integrative taxonomy consisted of six graduate-level students and took place over 4 weeks during January–February 2024 at the Department of Integrative Taxonomy and Biodiversity of Insects at the University of Hohenheim in Stuttgart, Germany. Each week focused on a different aspect of integrative taxonomy spanning “first discoveries” during specimen curation to writing a first draft taxonomic and phylogenetic manuscript. The timeline of the course was as follows: (1) week one provided background for the case study and focused on teaching the students curation and identification techniques for ants; (2) week two focused on DNA extraction and the UCE pipeline as well as applying previously assembled UCE data in species-delimitation analyses (Gray, 2023); (3) week three focused on morphometrics and traditional morphology-centered taxonomy as well as combining these results with the phylogenetic results; (4) week four focused on the writing process for peer-reviewed taxonomic and phylogenetic studies with the goal of producing a first draft of this manuscript. This course also included a workshop at the Stuttgart Museum of Natural History to instruct students on imaging techniques for arthropods as well as additional phylogenomic methods for species delimitation. The student cohort collaboratively generated the morphometric datasets and used previously generated UCE and COI data to infer the molecular phylogenies (Gray, 2023). Following this, the course instructors validated the results by independently generating the morphometric dataset and redoing phylogenetic analyses. The students wrote the first draft manuscript of this study that was revised by the instructors and students collaboratively. At the end of the course, students completed an anonymous survey and rated their agreement with the following statement designed to assess scientific self-efficacy: “I feel more capable of taking on my own projects related to the subject of the course.”

### Examined material

The specimens examined in this study have been deposited in the collections below:

CRC: Christian Rabeling Collection, University of Hohenheim, Stuttgart, Baden-Württemberg, Germany.
MCZC: Museum of Comparative Zoology Collection, Harvard University, Cambridge, Massachusetts, USA.

This study of the Vanuatuan *Pheidole sexspinosa* complex is primarily based on specimens collected *via* leaf litter sampling in 2011 and 2012 from Efate and Espiritu Santo by Christian Rabeling, Simone Cappellari-Rabeling, and the late Edward O. Wilson. These specimens are currently held in the repository of the Christian Rabeling Collection (CRC) at the University of Hohenheim, Stuttgart, Germany (Supplemental Information 1, Data S1). Additional specimens were acquired from the Museum of Comparative Zoology Collection (MCZC) at Harvard University (Cambridge, Massachusetts, USA) that were collected by hand. All field sampling as part of this study was assisted and approved by the government of Vanuatu. The dataset was also supplemented by occurrence records from primary literature and AntWeb (AntWeb, 2026). We used regional keys to aid in the identification of specimens that focus on the ants of Fiji (Sarnat & Economo, 2012), Polynesia (Wilson & Taylor, 1967), Micronesia (Clouse, 2007), and Australia (Shattuck, 2000). We also used images from AntWeb to aid in the identification of specimens (AntWeb, 2026). We used ArcGis Pro version 3.4.0 to make the regional map (ESRI, 2025) (Fig. 1).

### Species delimitation criteria

We used qualitative morphological traits such as degree of sculpturing, color, head shape, and spine shape to first posit morphospecies hypotheses for the Vanuatuan *Pheidole sexspinosa* species complex. We then tested these morphospecies hypotheses by combining quantitative morphometrics with phylogenomic evidence utilizing UCEs to infer a population-level phylogeny as well as to test for gene flow across morphospecies. We adhere to the Unified Species Concept, which defines species as separately evolving metapopulation lineages and treats reproductive isolation, diagnosability and monophyly as lines of evidence rather than defining criteria (De Queiroz, 2005). Accordingly, our final species decisions are based on the cumulative weight of all available evidence.

### Morphological measurements and indices

**Figure 3:**
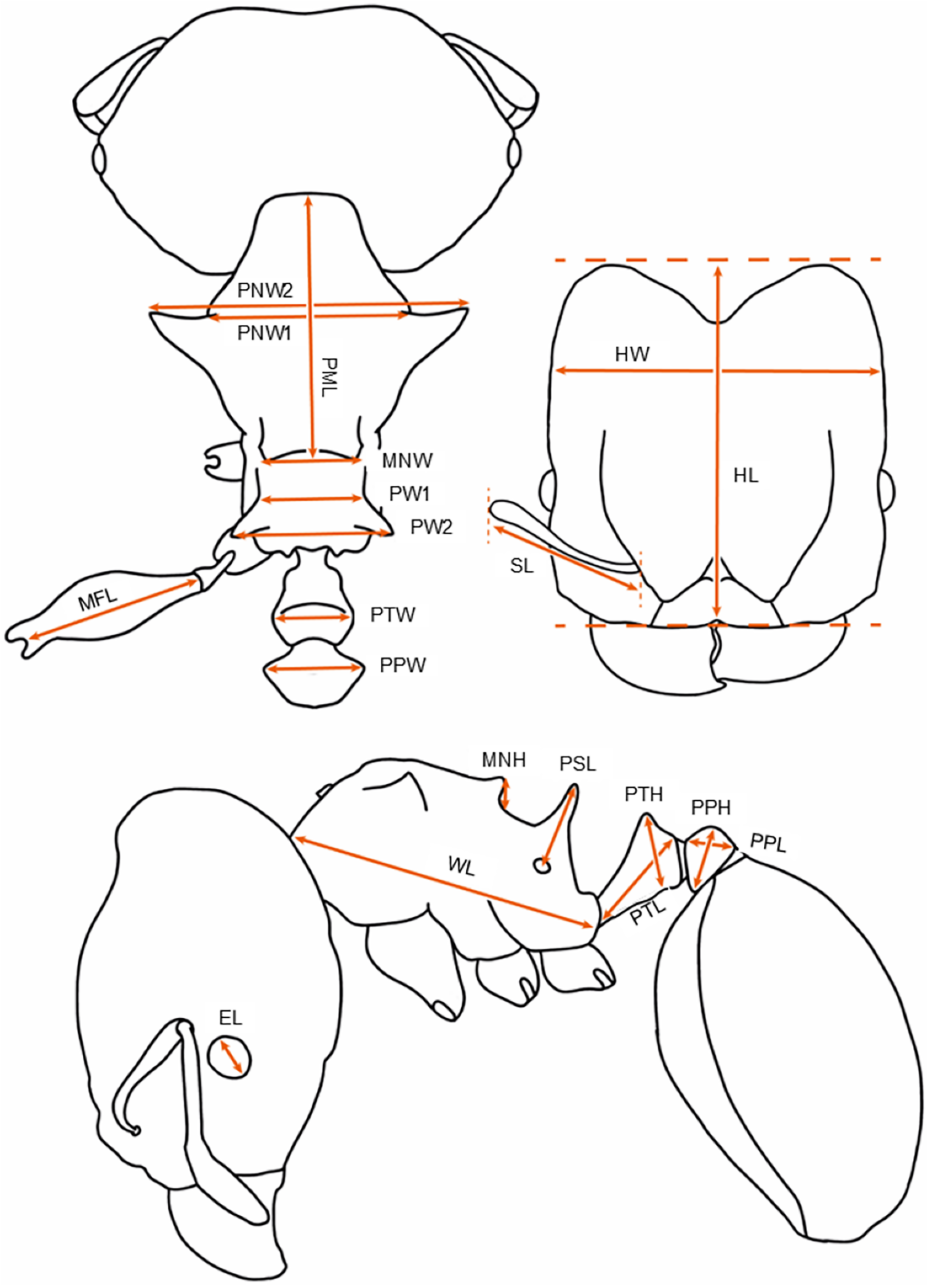
Illustration of a major worker in the *Pheidole sexspinosa* complex visualizing the 20 measurements taken for the morphometric analyses in this study (orange lines).

We measured 20 morphological traits for 51 Vanuatuan *Pheidole sexspinosa* complex workers (33 minor workers & 18 major workers) from 13 populations across three islands (Supplemental Information 2, Data S2). These 20 traits included those that were measured for the most recent taxonomic study of the *P. sexspinosa* complex that focused on Singaporean populations (Wang, Yamada & Eguchi, 2018), as well as additional traits we deemed potentially useful in differentiating the species. The measured specimens included at least one minor and major worker for each population when possible. The morphological measurements were taken using a Leica M125 stereo microscope equipped with an ocular micrometer lens. All measurements are presented in millimeters rounded to the hundredth (raw morphological data in µm; Supplemental Information 2, Data S2). Abbreviations and definitions for each trait are provided below (Fig. 3).

In frontal view:

HL: Head length: In minors, maximum distance from the midpoint of the anterior clypeal margin to the midpoint of the tangent between the posterior most projection of the vertex. In majors, measured from the midpoint of the tangent between the anterior most position of the clypeus to the midpoint of the tangent between the posterior most projection of the vertex.
HW: Head width: Measured at the widest point of the head behind eye level.
SL: Scape length: Maximum scape length, excluding basal condyle and neck.

In lateral view:

EL: Eye length: Maximum diameter of the eye measured in oblique lateral view.
WL: Weber´s length: Diagonal length of the mesosoma from the anterior point of the pronotal slope excluding the neck to the posteroventral margin of the propodeum.
MNH: Mesonotal spine height: Orthogonal distance from the apex of the mesonotal spine to the intersection with the mesonotal declivity or propodeum.
PSL: Propodeal spine length: Maximum length of the propodeal spine measured from the center of the propodeal spiracle.
PTL: Petiole length: Maximum diagonal length of petiole from the most anteroventral point of the peduncle to the most posterodorsal point at the junction to first helical tergite.
PTH: Petiolar node height: Maximum height of the petiolar node measured from the highest point of the node orthogonally to the ventral outline of the node.
PPL: Postpetiole length: Maximum length of the postpetiole from the anterior most point of the dorsal slope to the posterior juncture of the postpetiole and second helical tergite.
PPH: Postpetiole height: Maximum height of the postpetiole from the highest point of the node to the lowest point of the ventral process.

In dorsal view:

MNW: Mesonotal width: Maximum width of the mesonotum.
PML: Promesonotal length: Measured from the midpoint of anterior margin of the pronotal collar to the midpoint of a transverse line spanning the posterior-most points of mesonotal spines.
PNW1: Pronotal width excluding spines: Maximum width of the pronotum spanning from the bases of the pronotal spines.
PNW2: Pronotal width including spines: Maximum width of the pronotum spanning the apices of the pronotal spines.
PW1: Propodeal width excluding spines: Maximum distance between the bases of the propodeal spines.
PW2: Propodeal width including spines: Maximum distance between the apices of the propodeal spines.
PTW: Petiolar node width: Maximum width of the petiolar node.
PPW: Postpetiole width: Maximum width of postpetiolar node.
MFL: Metafemur length: Measured from the junction with the trochanter to the junction with the tibia.

Based on the measurements above, we estimated the following indices:

CI: Cephalic index: HW/HL * 100.
PSLI: Propodeal spine length index: PSL/HW * 100.

### Morphometric analyses

To test the species boundaries using a quantitative morphological framework, we applied a multivariate ratio analysis (MRA) that allows for the interpretation of a principal component analysis (PCA) in terms of trait ratios (Baur & Leuenberger, 2011), which assesses the differences in both size and shape of body measurements. All morphometric analyses considered minor and major workers separately. We followed MRA pipelines used in previous studies to disentangle cryptic species complexes in the Hymenoptera (Baur et al., 2014; Baur & Leuenberger, 2011). For the MRAs, we first calculated an isometric size axis (hereafter referred to as “isosize”) defined as the geometric mean of all traits. We then used a shape PCA (PCA using trait ratio space) to determine how well the quantitative trait ratios translate to distinct morphological clusters. To assess the amount of allometric variation in the dataset, we plotted isosize against shape PC1. If there is a significant and strong correlation between isosize and shape PC1, there is strong allometric variation present (Baur & Leuenberger, 2011). Lastly, we applied a type of linear discrimination analysis (LDA) in the MRA framework that finds trait ratios that maximizes the discrimination between morphological clusters, which is referred to as the “LDA ratio extractor.” We then compared these LDA extracted ratios to commonly used indices in taxonomic studies of ants that generally focus on ratios of the same body part such as only head or petiole measurements. The R programming language and environment was used for statistical computing and data analysis (R Core Team, 2025). We used modified R-scripts provided by Baur & Leuenberger (2020) to perform the MRA pipeline.

### Taxon sampling for UCEs

In total, ten worker specimens consisting of both minors and majors of the Vanuatuan *Pheidole sexspinosa* complex from the islands Efate and Espiritu Santo were included in the taxon sampling to infer a population-level phylogeny (Supplemental Information 3, Data S3). In addition, we included two workers of the tramp species *Pheidole megacephala* (Fabricius, 1793) as the outgroup for the phylogenetic analyses. Most specimens were preserved in 100% ethanol solution stored at −80 °C but there were populations only represented by point-mounted material.

### Molecular data generation and UCE matrix assembly

The UCE data used in this study was generated and assembled as part of a broader phylogenomic study of Vanuatuan ants (Gray, 2023). In brief, established protocols to extract and amplify UCEs for ants were followed (Branstetter et al., 2017). DNA was extracted using a DNeasy Blood and Tissue Kit (Qiagen, Hilden, Germany) non-destructively from adult workers to retain voucher specimens. For each sample, DNA was quantified using a Qubit fluorometer with the high-sensitivity kit (Life Technologies, Inc., Carlsbad, CA, USA) then sheared to a target size of approximately 400–600 bp. For specimens collected after the year 2000, shearing settings were set to 1-min shearing time, 25% amplitude, and cycles of 10 s on and 10 s off. For samples collected before the year 2000, shearing settings were set to 20 s shearing time, 25% amplitude, and cycles of 10 s on and 10 s off. The sheared DNA was used as input for a modified genomic DNA library preparation protocol (Kapa Hyper Prep Library Kit; Kapa Biosystems, Wilmington, MA, USA), closely following Branstetter et al. (2017) using TruSeq-style dual indexing adapters (Glenn et al., 2019). After library preparation, samples were enriched further and UCEs captured using the Hym 2.5Kv2A RNA probe ant-specific bait at 0.1X concentration (Daicel Arbor Biosciences, Arbor, MI, USA), which aims to capture 2,524 UCE loci (Branstetter et al., 2017). Enriched samples were then combined into sets of pools with equal molar concentrations per sample, dehydrated, then rehydrated to produce approximately 3.4 μL of pooled library with a target concentration of 147 ng/μL. Samples were sent to Novogene Corporation Inc., (Sacramento, CA, USA) for HiSeq PE150 sequencing and demultiplexing.

The processing of the demultiplexed reads was done using the PHYLUCE v1.7.3 pipeline (Faircloth, 2016), which included trimming adapter contamination and low-quality bases using Illumiprocessor (Faircloth et al., 2013), assembling contigs using SPAdes v3.15.3 (Bankevich et al., 2012), aligning the resulting FASTA files of individual samples with MAFFT using the no trim option (Katoh, Asimenos & Toh, 2009), and instead trimmed UCE loci using Gblocks (settings: b1 = 0.5, b2 = 0.5, b3 = 12, b4 = 7) (Castresana, 2000). For downstream analyses, individual UCE locus alignments that included 90% taxon-completeness were used (hereafter referred to as the “90p” dataset). Summary statistics for each alignment were estimated using AMAS (Borowiec, 2016).

### Phylogenetic analyses of UCEs

The phylogenetic analyses were done by the students and then redone by the instructors. We partitioned the 90p UCE dataset for phylogenetic analysis using the Sliding-Window Site Characteristics based on Entropy method (SWSC-EN) specifically designed for UCEs (Tagliacollo & Lanfear, 2018). The SWSC-EN partitioning method incorporates the architecture of UCEs where each locus is broken up into its three parts, being the conserved core region and the two flanking regions that become more variable with distance from the conserved core. We then used the resulting SWSC-EN partitioning schemes to infer a phylogeny using the maximum-likelihood framework in IQ-TREE 2 (Minh et al., 2020). We allowed for each partition to have its own rate of evolution and tested for the best-fit model of evolution for each partition using Modelfinder2 with the merging of similar partitions (option-m MFP+MERGE; rcluster algorithm to check top 10% of merging schemes). To assess branch support, we performed 1,000 replicates of the ultrafast bootstrap approximation (=“UFB”) where we considered ≥95% as strong support.

### Phylogenetic analyses of COI region

Due to the presence of mitochondrial DNA in UCE sequences as an unintended byproduct of target enrichment (Longino & Branstetter, 2021; Ströher et al., 2017), we performed a separate and complementary analysis of Cytochrome C Oxidase Subunit I (COI) sequence data to phylogenetically contextualize the Vanuatuan *Pheidole sexspinosa* complex with other regional populations for which COI data is publicly available on NCBI Genbank (https://www.ncbi.nlm.nih.gov/genbank/). To extract COI sequences from our UCEs, we first downloaded a COI sequence from NCBI GenBank referred to as *Pheidole sexspinosa* (GenBank accession number: MH726209.1; locality: Singapore) (Wang, Yamada & Eguchi, 2018). We then used this sequence as a probe for a PHYLUCE v1.7.3 function to extract regions from the best matching contigs to the COI probe sequence (“phyluce_assembly_match_contigs_to_barcodes”). This function also runs the results against the “barcode of life data system” (“BOLD”) for each sequence, which is useful for checking species ID and searching for potential contamination (Ratnasingham & Hebert, 2007). We combined all available *Pheidole sexspinosa* complex COI sequences from GenBank with our extracted COI sequences (only sequences with ≥500 bp were included), aligned the dataset using MAFFT-L-INS-I (Katoh, Asimenos & Toh, 2009), then imported the file to AliView (Larson, 2014) to inspect the alignment for anomalies such as indels or highly divergent sequences. The resulting alignment file was then used as input to infer a phylogeny using two methods. First, we inferred a maximum-likelihood phylogeny in IQ-TREE 2 using the GTR model of sequence evolution and 1,000 replicates of ultrafast bootstrap approximation to assess branch support (=“UFB”). Second, we inferred a neighbor-joining phylogeny using the p-distance method with uniform rates among sites in MEGA11 (Tamura, Stecher & Kumar, 2021) and 1,000 replicates of bootstrap approximation to assess branch support (=“BS”). For both phylogenetic inference methods, we considered ≥95% UFB/BS as strong support.

### Testing for gene flow using 3s

Using the software package 3s with the UCE data and the resulting phylogenetic tree topology as input, we tested for gene flow within and between the Vanuatuan morphospecies under the isolation-with-migration model (Dalquen, Zhu & Yang, 2017; Zhu & Yang, 2012). The list of the triplets, phylogenetic configurations, and critical values are provided in the results section (Table 1).

**Table 1:** Results of six 3s gene flow analyses under the isolation-with-migration model of the Vanuatuan *Pheidole sexspinosa* complex with outgroup specimens *P. megacephala*. The “Configuration” column reflects the *a priori* species hypotheses under no gene flow.

Combination	Island, locality	Loci shared	Configuration	Critical value	2DInL	H0	Gene flow
*P. epaoensis* (VAN218)	Efate, 3 km W Epao	2,106	1	M1 = 5.99	−475.82	Not rejected	**No**
*P. tanakarensis* (VAN220)	Espiritu Santo, Cons. Site		3	M2 = 9.49	−1,275.27		
*P. nivanuatu* (VAN235)	Espiritu Santo, Mount Saratsi		2				
*P. megacephala* (VAN202)	Efate, 13 km W Port Vila	2,021	2	M1 = 2.71	19.40	Rejected	**Yes**
*P. epaoensis* (VAN218)	Efate, 3 km W Epao		1	M2 = 7.82	23.80		
*P. epaoensis* (VAN219)	Efate, “Worowloa”		1				
*P. nivanuatu* (VAN235)	Espiritu Santo, Mount Saratsi	1,979	1	M1 = 2.71	60.91	Rejected	**Yes**
*P. nivanuatu* (VAN237)	Espiritu Santo, Mount Voutemele		1	M1 = 7.82	91.25		
*P. nivanutu* (VAN238)	Espiritu Santo, Cons. Site		1				
*P. tanakarensis* (VAN220)	Espiritu Santo, Cons. Site	2,038	1	M1 = 2.71	14.77	Rejected	**Yes**
*P. tanakarensis* (VAN221)	Espiritu Santo, Mount Saratsi		1	M1 = 7.82	22.42		
*P. tanakarensis* (VAN223)	Espiritu Santo, Mount Voutemele		1				
*P. megacephala* (VAN201)	Ambrym, 5.6 km NE Port Vato	2,049	3	M1 = 5.99	3,067.17	Not rejected	**No**
*P. nivanuatu* (VAN238)	Espiritu Santo, Cons. Site		1	M2 = 9.49	−424.44		
*P. tanakarensis* (VAN220)	Espiritu Santo, Cons. Site		2				
*P. epaoensis* (VAN218)	Efate, 3 km W Epao	2,026	3	M1 = 5.99	−0.001	Not rejected	**No**
*P. nivanuatu* (VAN236)	Espiritu Santo, Mount Tanakar		1	M2 = 9.49	1.73		
*P. tanakarensis* (VAN222)	Espiritu Santo, Mount Tanakar		2				

### Imaging of specimens

Composite images were generated using a Leica K5C digital camera mounted to a Leica M205 C stereomicroscope and assembled using Leica Application Suite (version 5.3.0.).

The electronic version of this article in Portable Document Format (PDF) will represent a published work according to the International Commission on Zoological Nomenclature (ICZN), and hence the new names contained in the electronic version are effectively published under that Code from the electronic edition alone. This published work and the nomenclatural acts it contains have been registered in ZooBank, the online registration system for the ICZN. The ZooBank LSIDs (Life Science Identifiers) can be resolved and the associated information viewed through any standard web browser by appending the LSID to the prefix http://zoobank.org/. The LSID for this publication is: urn:lsid:zoobank.org:pub:BAFC74A4-2BC1-451E-98CD-2B888B28C6DB. The online version of this work is archived and available from the following digital repositories: PeerJ, PubMed Central SCIE and CLOCKSS.

## Results

### Integrative taxonomic species delimitation supports the presence of three distinct species in the Vanuatuan *Pheidole sexspinosa* complex

**Figure 4:**
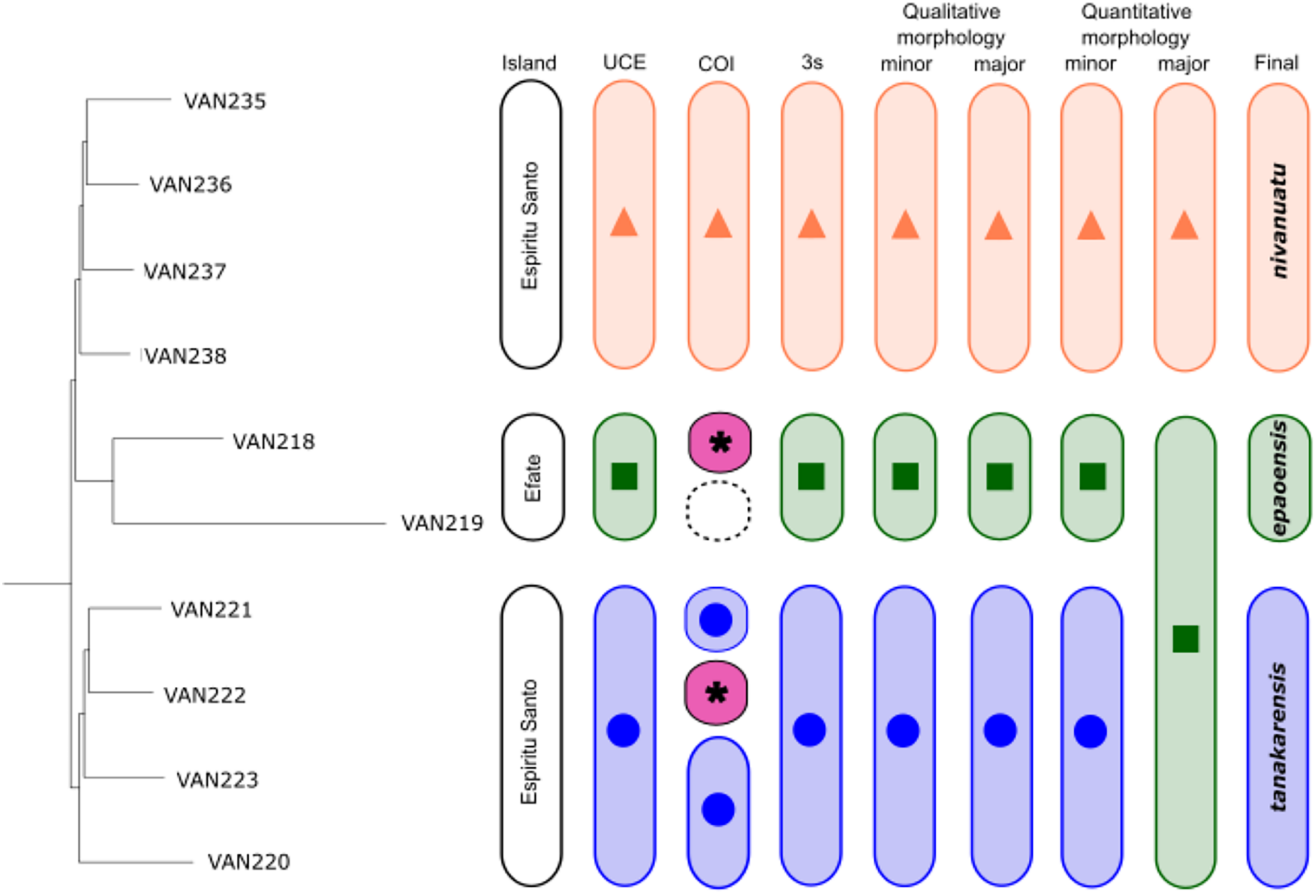
Summary of the results from our integrative taxonomic analyses of the Vanuatuan *Pheidole sexspinosa* complex where each shape/color represents species boundaries. The phylogeny shown here is from the SWSC-EN partitioned analysis with the sample code given for each tip (see Fig. 7 for details). The “Island” column shows which island the samples were collected from in the phylogeny. The “UCE” & “COI” columns refer to the presence of monophyletic groups in the SWSC-EN and COI phylogenies, respectively. The “3s” column refers to groups of isolated gene pools. The “Qualitative morphology” and “Quantitative morphology” sections refer to morphological species supported by distinct characters and morphospace, respectively. Our final species delimitation decisions are shown in the “Final” column. See the “Taxonomic Results” section for the formal descriptions and diagnoses of each species.

When integrating the results from the morphometric and phylogenomic analyses considering both minor and major workers, there are three well-supported species in the Vanuatuan *P. sexspinosa* complex, which we describe below as *P. epaoensis* **sp. nov.**, *P. nivanuatu* **sp. nov.**, and *P. tanakarensis* **sp. nov.** (Fig. 4). Each species is morphologically distinguishable, monophyletic and phylogenetically distinct. In addition, the species show no signals of gene flow between them. See the following results sections for the individual analyses and taxonomic descriptions. The results were consistent between student and instructor datasets.

### Morphometrics support the presence of three distinct morphological species

**Figure 5:**
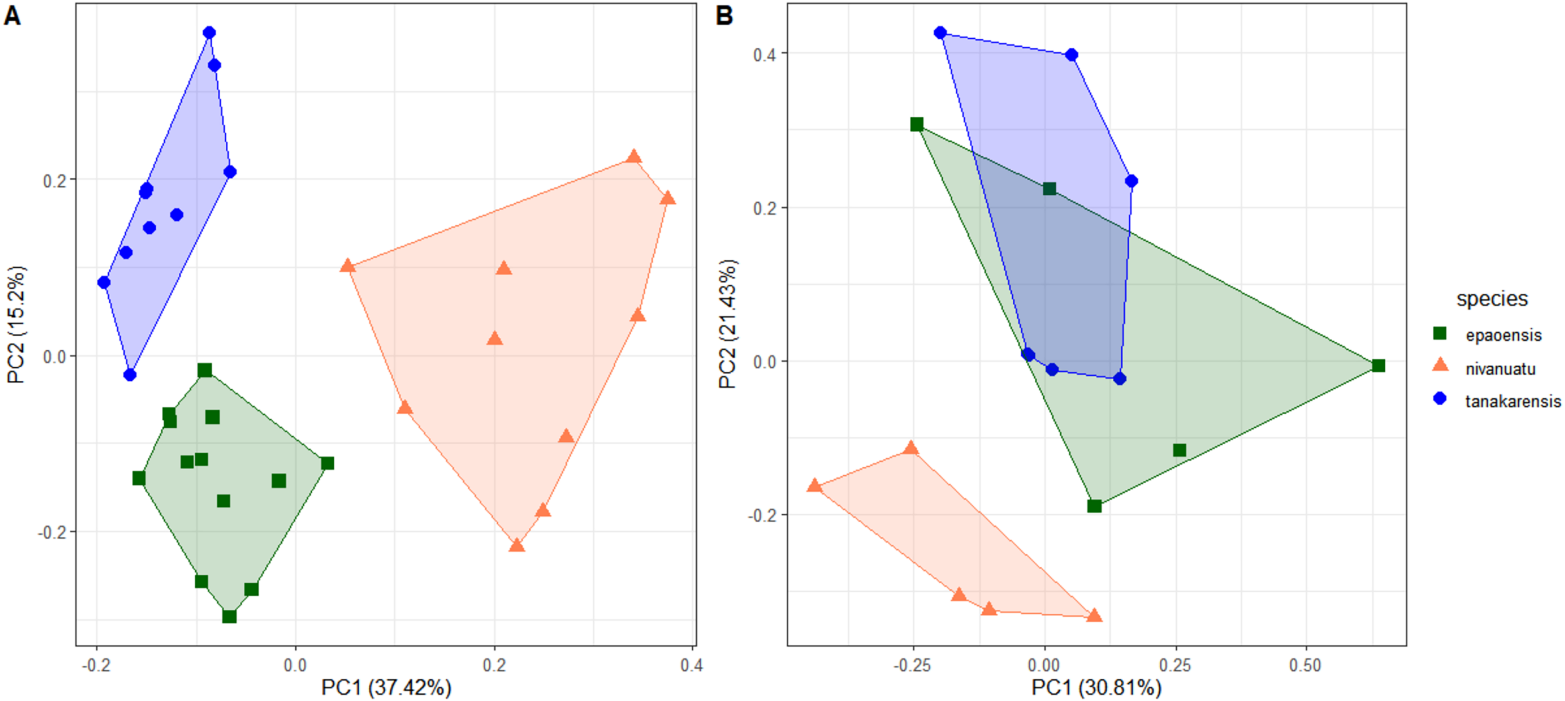
Shape PCAs using 20 measured traits for minor workers (A) and major workers (B) in the Vanuatuan *Pheidole sexspinosa* complex. *Pheidole epaoensis* is represented by green squares, *P. nivanuatu* by orange triangles, and *P. tanakarensis* by blue circles.

The shape PCA using the 20-trait dataset supported the presence of three distinct morphological species based on the morphospace of the minor workers (Fig. 5A). When plotting isosize *vs*. shape PC1 in the minor workers, there was a moderate but significant correlation (Adjusted R^2^ = 0.67, *p*-value = 7.343e−09) suggesting the presence of a moderate amount of allometric variation (Supplemental Information 4, Fig. S1). For the major workers, the shape PCA showed much overlap between *P. epaoensis* and *P. tanakarensis* but *P. nivanuatu* was distinct (Fig. 5B). When plotting isosize *vs*. shape PC1 in the major workers, there was no significant correlation (Adjusted R^2^ = 0.024, *p*-value = 0.257) suggesting a negligible amount of allometric variation (Supplemental Information 4, Fig. S2).

**Figure 6:**
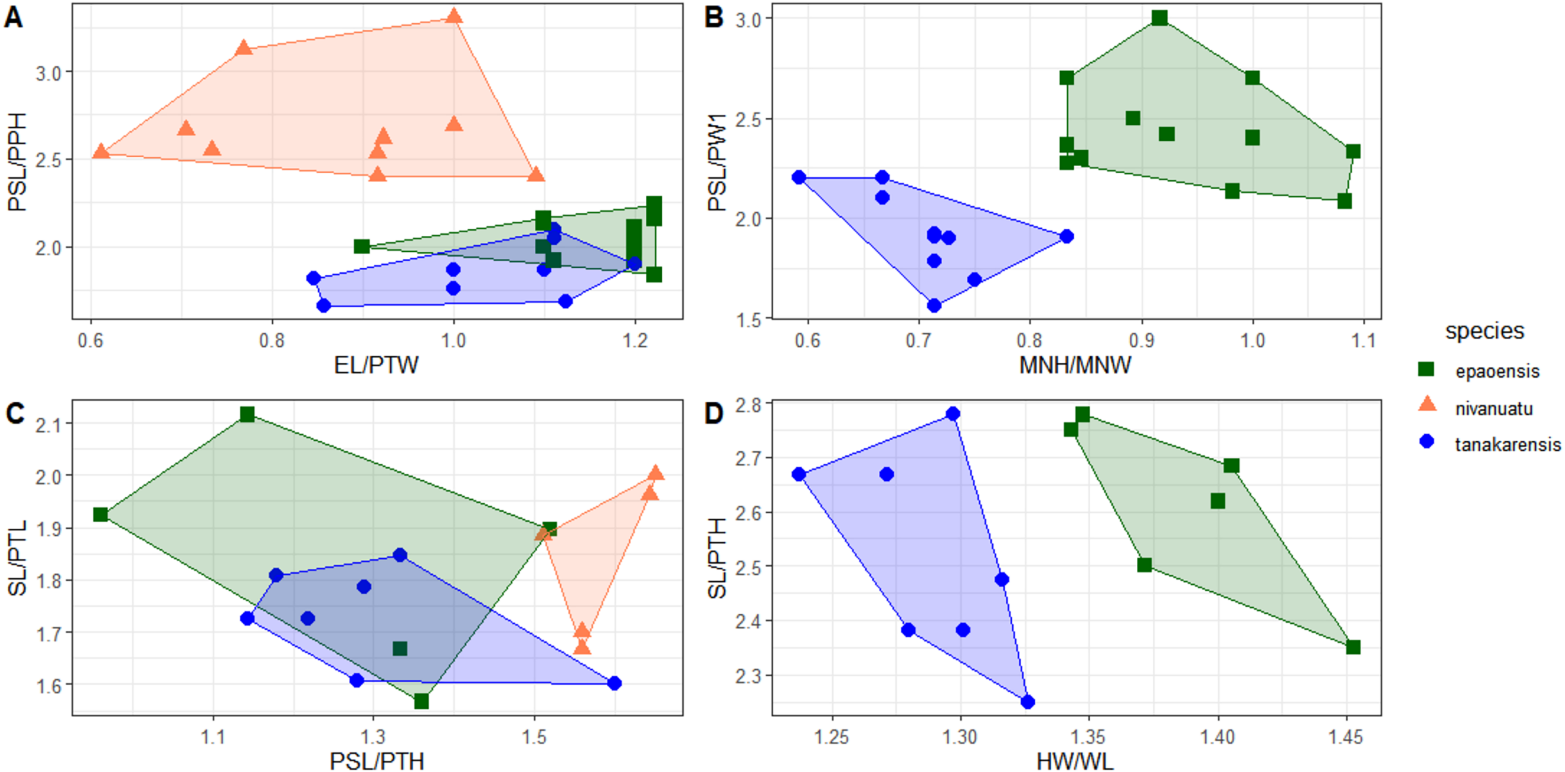
LDA ratio extractor plots of the Vanuatuan *Pheidole sexspinosa* complex comparing minor workers (A & B) and major workers (C & D). Pair-wise species comparison between *P. epaoensis* and *P. tanakarensis* in minor workers (B) and major workers (D). *Pheidole epaoensis* is represented by green squares, *P. nivanuatu* by orange triangles, and *P. tanakarensis* by blue circles.

Based on the LDA ratio extractor using all 20 traits for the minor workers only, EL/PTW *vs*. PSL/PPH was the most discriminatory trait ratio comparison to differentiate the Vanuatuan *P. sexspinosa* complex species (Fig. 6A). With this trait ratio comparison, there was still an overlap between minor workers of *P. epaoensis* and *P. tanakarensis*. To maximize the potential morphospace differences between species in the LDA ratio extractor analysis, it is recommended to do pair-wise comparisons (Baur & Leuenberger, 2011). Therefore, when comparing the minor workers of *P. epaoensis* and *P. tanakarensis* only, PSL/PTL *vs*. MNH/MNW was the most discriminatory trait ratio comparison (Fig. 6B). For the major workers, there were numerous traits that were weakly or negatively correlated with other traits, which cause errors in the LDA ratio extractor and need to be excluded. When including only the traits HW, HL, SL, WL, PSL, PTL, PTH, PTW, and MFL, it resulted in the most discriminatory trait ratio comparison of PSL/PTH *vs*. SL/PTL, although there was still a significant overlap between major workers of *P. epaoensis* and *P. tanakarensis* (Fig. 6C). When performing the pair-wise species comparison between major workers of *P. epaoensis* and *P. tanakarensis* only with traits HW, HL, SL, WL, PTL, PTH, PML, and PTW, the most discriminatory trait ratio was HW/WL *vs*. SL/PTH, which resulted in no overlap (Fig. 6D). Based on the CI and PSLI indices, there was significant overlap between *P. epaoensis* and *P. tanakarensis* but *P. nivanuatu* was distinct for both minor and major workers (Supplemental Information 4, Figs. S3 & S4). Therefore, the results of the LDA ratio extractor analysis and traditional index comparisons were comparable, except when performing pair-wise species comparisons in which the LDA ratio extractor identified trait ratios to delimit the species more robustly.

### UCE dataset summary statistics

For the ten specimens used to infer a population-level phylogeny of the Vanuatuan *Pheidole sexspinosa* complex, we recovered a mean UCE contig coverage of 48× (range 34–63×) and a mean UCE contig length of 1,979 bp (range 1,126–2,342 bp). After the alignment, trimming, and filtering of the UCE dataset to 90% completeness (“90p” dataset), our UCE matrix consisted of 2,130 loci and 3,890,399 bp of which 85,867 bp were parsimony informative sites.

### UCEs support the presence of three phylogenetically distinct species with no evidence of gene flow

**Figure 7:**
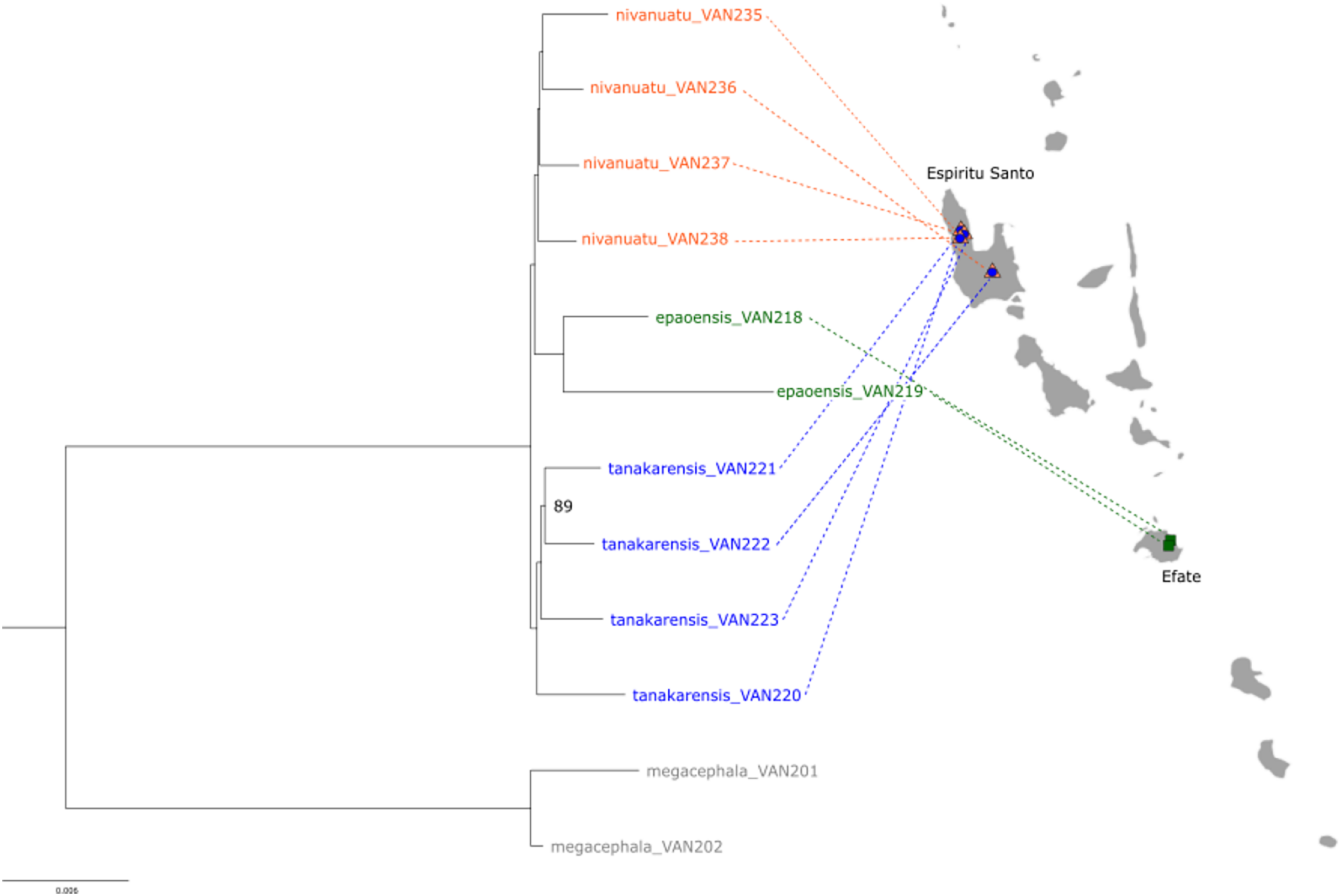
SWSC-EN phylogeny based on 2,130 UCE loci of the Vanuatuan *Pheidole sexspinosa* complex showing sampling localities for each tip. Each of the species is shown by a distinct color/symbol with *P. nivanuatu* as orange/triangle, *P. epaoensis* as green/square, and *P. tanakarensis* as blue/circle. All non-labeled nodes have ≥95 UFB support.

After applying the SWSC-EN partitioning method, the 2,130 UCE loci were split into 6,390 partitions. The topology of the SWSC-EN phylogeny supported the presence of three distinct monophyletic species (Fig. 7). The sympatric species *P. tanakarensis* and *P. nivanuatu* on Espiritu Santo each form reciprocally monophyletic clades with the allopatric Efate species *P. epaoensis* inferred as sister to *P. nivanuatu*.

Using the UCE data as input, the 3s analysis detected intraspecific gene flow between populations of *P. epaoensis*, *P. nivanuatu*, and *P. tanakarensis*. However, there was no interspecific gene flow detected under the isolation-with-migration model, even for the populations that occur in direct sympatry on Espiritu Santo such as *P. tanakarensis* and *P. nivanuatu* (Table 1).

### COI phylogeny supports *in situ* diversification in the Vanuatuan *Pheidole sexspinosa* complex

**Figure 8:**
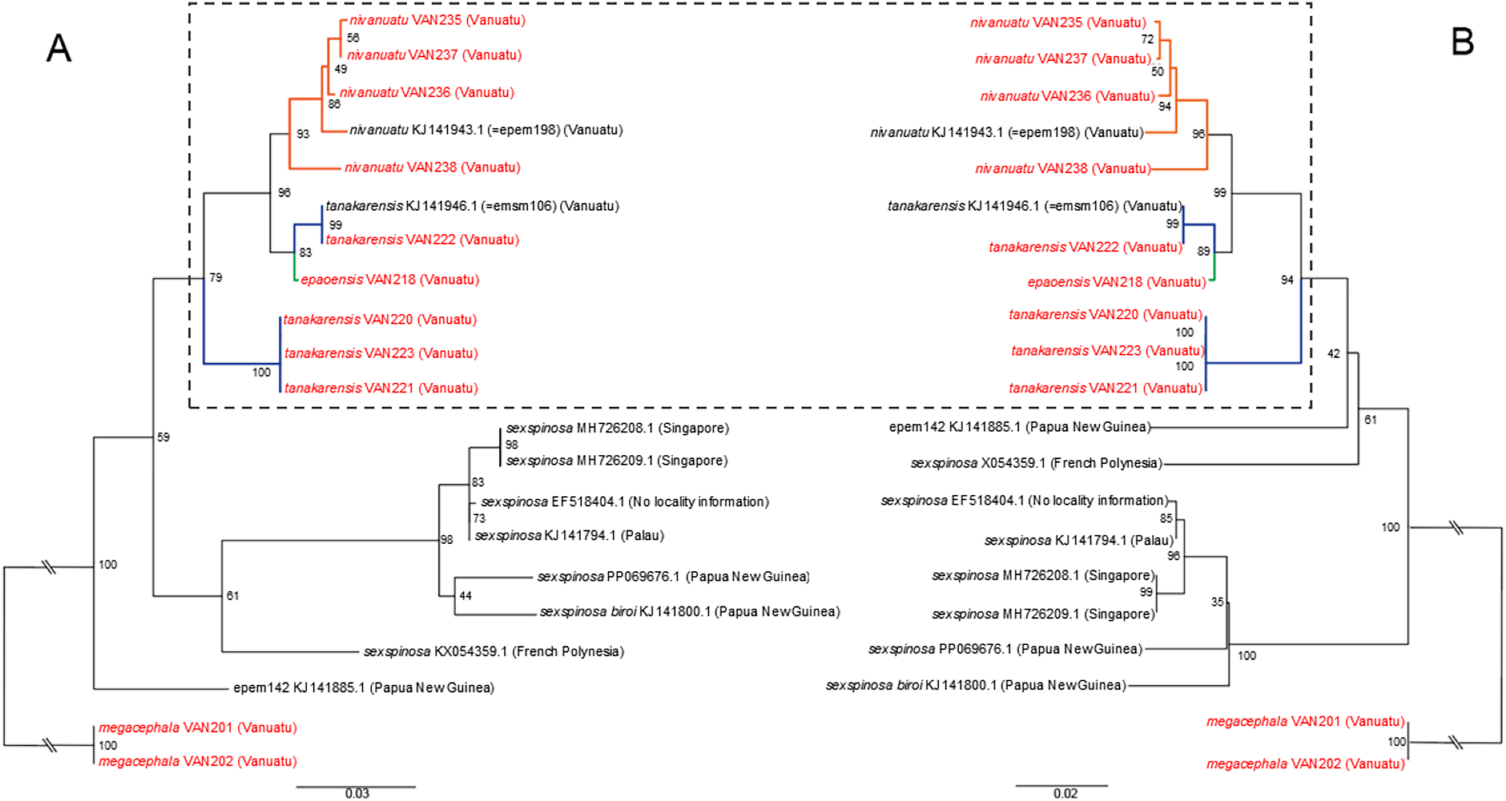
Maximum likelihood (A) and neighbor joining (B) COI phylogenies with branch supports labeled and the locality information for each tip. The dashed box indicates the monophyletic Vanuatuan *Pheidole sexspinosa* complex. *Pheidole nivanuatu* is represented by orange branches, *P. epaoensis* by green branches, and *P. tanakarensis* by blue branches. Tip labels in red are COI sequences extracted from UCEs in this study. Tip labels in black are COI sequences integrated from NCBI GenBank with the associated accession number. Non-italicized morphospecies names correspond to those used in Economo et al. (2015).

After extracting the COI sequences from the UCE samples, the resulting alignment consisted of 21 samples and was 642 bp in length. The only sample from the UCE dataset that was excluded from the COI dataset was a sample of *P. epaoensis* (VAN219) due to its short contig length from the barcode-to-contig matching analysis (<500 bp). The COI phylogenies reflected similar findings to the previous phylogenetic study of Old World *Pheidole* that included some populations of the *P. sexspinosa* complex (Economo et al., 2015; Fig. 2 this study), namely in that the southeastern Asian populations of *P. sexspinosa* and *P. s. biroi* that occur in Singapore, Palau, and New Guinea formed a well-supported clade separate from the Vanuatuan populations (Fig. 8; UFB = 98/BS = 100). In addition, the Vanuatu members of the *P. sexspinosa* complex were recovered as monophyletic in both phylogenetic inference methods but with moderate support (Fig. 8 shown with dashed box; UFB = 79/BS = 94). Within Vanuatu, the only species that was inferred as monophyletic was *P. nivanuatu* with moderate support (Fig. 8 with orange branches; UFB = 93/BS = 94).

## Taxonomic results

### Diagnosis of the *Pheidole sexspinosa* complex modified from Wang, Yamada & Eguchi (2018) to include the Vanuatuan species

In major workers, posterior margin of head in full-face view with deep median concavity. Dorsal and lateral faces of posterolateral corner reticulate with interspaces micro-reticulate and shining. Frons longitudinally rugose with interspaces microreticulate and shining. Antennal scrobe present and conspicuous beneath frontal carina. Hypostoma without median process but with subtriangular processes laterally. Antennae 12 segmented with three segmented club. Presence of three pairs of dorsal spines. Pronotal spines linear and directed anterolaterally. Mesonotal spines present and variable in size from a denticle and subtriangular horn to broadly-fused rounded spines. Propodeal spines linear. Mesopleuron and metapleuron smooth and shiny to rugoreticulate. Petiolar node bilobed. Postpetiole expanded laterally to form acute angles to small tooth-like protrusions. Gaster with superficial reticulate sculpturing.

In minor workers, posterior margin of head in full-face view weakly to moderately concave. Preoccipital carina well developed as a low lamella which in full-face view forms a minute tooth-like protrusion at the posterolateral corner of head. Posterior and ventrolateral margin of head variable in sculpture from smooth and shiny to rugoreticulate. Frons sculpturing variable ranging between entirely smooth and shiny to major parts being longitudinally rugose. Antennae 12 segmented with three segmented club. Pronotum and mesonotum fused. Presence of three pairs of dorsal spines. Pronotal spines linear and directed anterolaterally. Mesonotal spines present and variable in size from small and acute to broadly rounded. Propodeal spines variable from linear to slightly curved. Dorsal and lateral faces of promesonotum variable in sculpturing ranging from entirely smooth and shiny to rugulose. Mesopleuron, metapleuron, and dorsal face of propodeum variable in sculpture ranging from smooth and shiny to rugoreticulate with interspaces smooth and shining. Gaster sculpturing variable ranging between finely sculptured to smooth and shiny. White/translucent patch, if present, restricted to first gastric tergite.

### Differential diagnosis of the Vanuatuan members of the *Pheidole sexspinosa* complex

A unique element shared amongst the Vanuatuan members of the *P. sexspinosa* complex is most readily observed in the minor workers, where the Vanuatuan species have much reduced sculpturing and fewer standing setae on the entire body surface compared to individuals from other populations of the *P. sexspinosa* complex. The lack of sculpturing is most readily seen on the frons as well as the entire lateral mesosoma. In most populations of the Vanuatuan species, minor workers possess sparse, appressed setae on the first gastric tergite rather than abundant standing to suberect setae. The only other population that lacks standing setae on the gaster appears to be a population of *P. s. biroi* from New Guinea (AntWeb specimen CASENT0904307), but this specimen has rugose sculpturing along the entire lateral mesosoma.

**Key to the minor workers of the *Pheidole sexspinosa* species complex in Vanuatu:**

*Pheidole sexspinosa* is a morphologically variable taxon with an extensive geographic range in the South Pacific region and the species complex is in dire need of revision. Based on prior taxonomic work and comparing images of “*P. sexspinosa*” available on AntWeb, it potentially comprises of multiple cryptic species. Furthermore, the holotype of *P. sexspinosa* is a heavily damaged major worker, preventing a meaningful comparison. Due to the distinctness of the Vanuatuan species and the lack of material needed to revise the taxonomy of the *P. sexspinosa* group properly, we decided to limit this key to the species that are endemic to Vanuatu.

**Figure 9:**
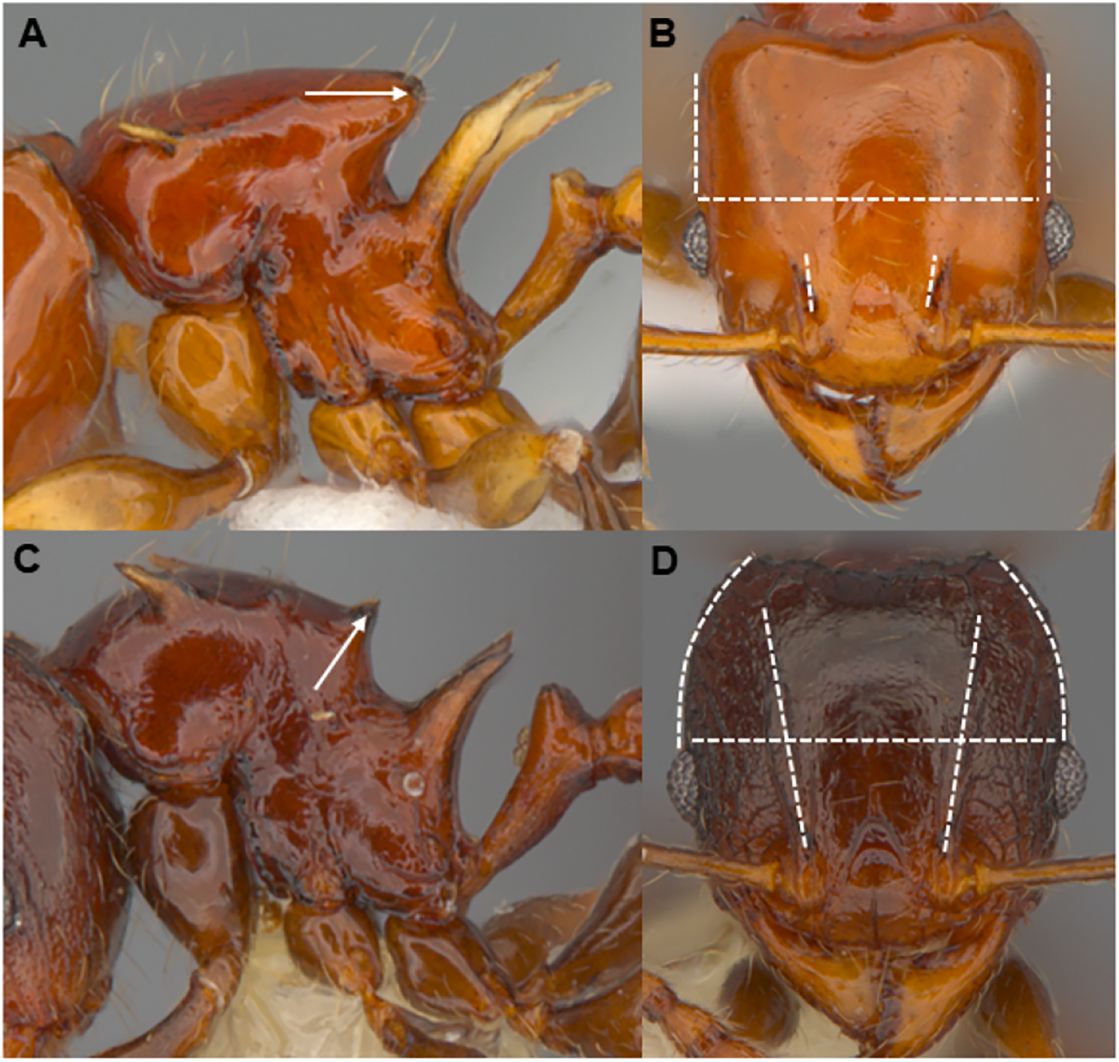
Mesonotal spine (A & C) and head shape comparisons (B & D) for the minor workers of *Pheidole nivanuatu* (A & B) and *Pheidole tanakarensis* (C & D).

(1) Head and mesosoma with orange-red coloration contrasting with lighter colored legs, propodeal spines, and gaster. Mesonotal spines blunt and rounded, pointed posteriorly (Fig. 9A). In frontal view, head shape subrectangular with lateral margins posterior of eye parallel or nearly so (Fig. 9B). Frontal carinae reduced, not surpassing posterior level of eyes (Fig. 9B). PSL/PPH > 2.40 ***nivanuatu* sp. nov.**
–Head and mesosoma brown to dark brown weakly contrasting with legs, propodeal spines, and gaster. Mesonotal spines sharp, pointed diagonally (Fig. 9C). In frontal view, head shape ovate with lateral margins tapering posteriorly (Fig. 9D). Frontal carinae extending to posterior margin of eyes (Fig. 9D). PSL/PPH < 2.40 2
2(1) In frontal view, frontal carinae surpass level of eyes then intersect with longitudinal sculpturing that extends towards the posterior margin of head. Frons with longitudinal ridges located medially from frontal carinae. MNH/MNW ≤ 0.83 ***tanakarensis* sp. nov.**
–In frontal view, frontal carinae barely reach level eyes or just surpass it without intersecting any longitudinal ridges. Frons smooth and shiny without longitudinal ridges located medially from frontal carinae. MNH/MNW ≥ 0.83 ***epaoensis* sp. nov.**

**Key to the major workers of the *Pheidole sexspinosa* species complex in Vanuatu:**

**Figure 10:**
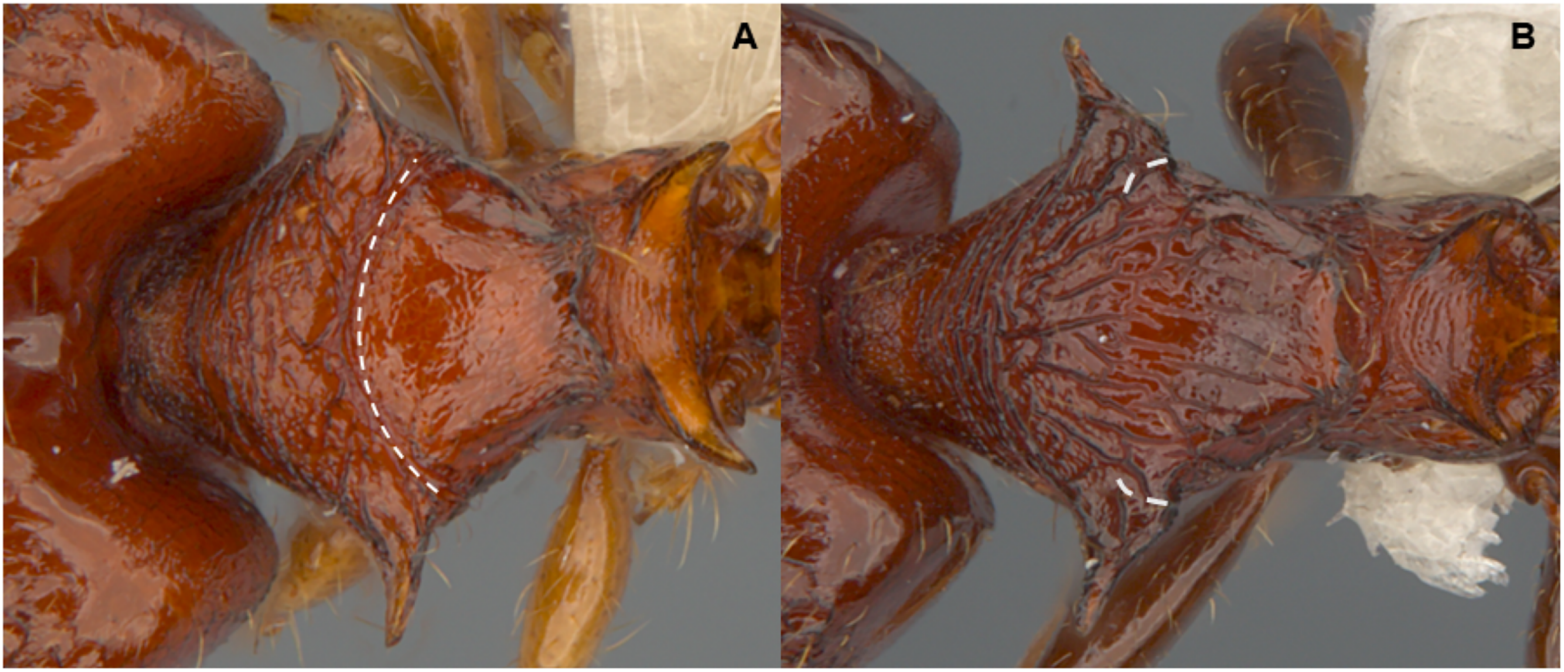
Mesonotal groove comparisons for the major workers of *Pheidole nivanuatu* (A) and *Pheidole tanakarensis* (B).

(1) Head and mesosoma orange-red contrasting lighter-colored legs and gaster. Pronotum and mesonotum separated by prominent continuous groove traversing entire mesonotum (Fig. 10A). Mesonotum smooth and shiny (Fig. 10A). PSL/PTH > 1.51 ***nivanuatu* sp. nov.**
–Head and mesosoma brown to dark brown weakly contrasting legs and gaster. Pronotum and mesonotum separated by discontinuous groove (Fig. 10B). Mesonotum with longitudinal ridges and reticulate sculpturing (Fig. 10B). PSL/PTH rarely > 1.51 2
2(1) Posterior half of pronotum with prominent longitudinal medial ridge that forks into two transverse ridges along mesonotal groove. Sculpturing on pronotum as transverse rugulae. Lateral margins of post petiole extend as subtriangular projections. Gaster uniformly brown lacking anterior white to yellowish patches. HW/WL > 1.34 ***epaoensis* sp. nov.**
–Posterior half of pronotum with longitudinal or radial ridges that extend towards lateral margins. Sculpturing on pronotum as longitudinal rugulae. If single medial ridge on pronotum is present, lateral margins of post petiole extend as acute to digitiform spines. Anterior half of gaster often with anterior white to yellowish patches. HW/WL < 1.34 ***tanakarensis* sp. nov.**

**Species accounts**

**Figure 11:**
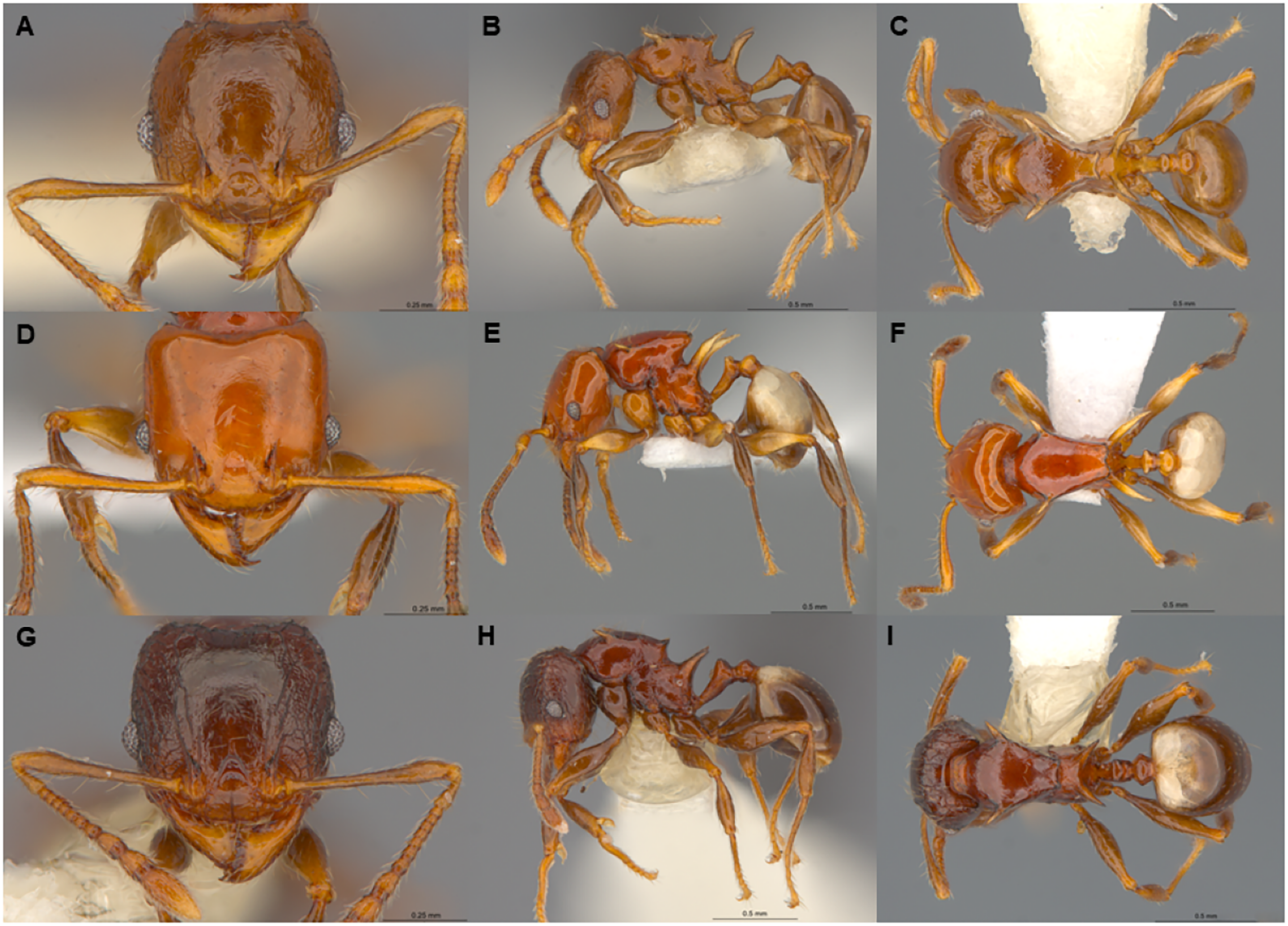
Comparison of holotype minor workers of *Pheidole epaoensis* (A–C), *P. nivanuatu* (D–F), and *P. tanakarensis* (G–I) in frontal (A, D, G), lateral (B, E, H), and dorsal (C, F, I) views. The scale bars represent 0.25 mm in A, D, G and 0.5 mm in all other images.

**Figure 12:**
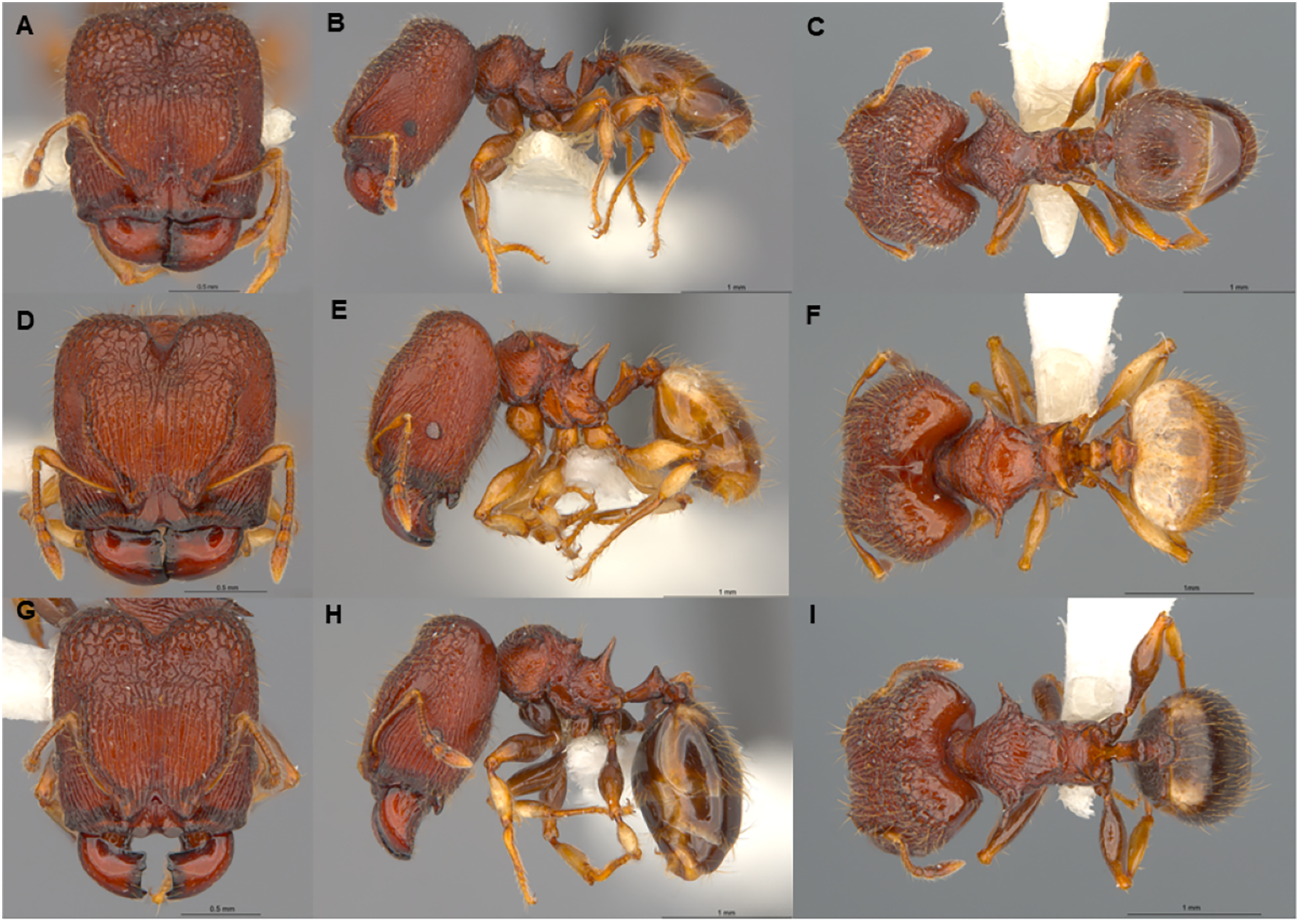
Paratype major workers of *Pheidole epaoensis* (A–C), *P. nivanuatu* (D–F), and *P. tanakarensis* (G–I) in frontal (A, D, G), lateral (B, E, H), and dorsal (C, F, I) views. The scale bars represent 0.5 mm in A, D, G and 1.0 mm in all other images.

***Pheidole epaoensis***, Gray, von Adelmannsfelden, Ahmed, Brandstetter, Alvarez, Härtel, Mahmood, Mera-Rodríguez & Rabeling **sp. nov.** (Figs. 11A–11C & 12A–12C)

[LSID urn:lsid:zoobank.org:act:3FF8A4DE-A18B-4671-9E4C-1ABDBC0E1659]

### Diagnosis

*Pheidole epaoensis* is currently only known from Efate and so far has not been collected in sympatry with any other *P. sexspinosa* complex species. This species is most readily identified by the minor workers based on the following combination of characters: the frons is smooth and shiny, whereas the lateral and dorsal parts of the head are covered with rugoreticulate sculpture; the frontal carinae are short, barely surpassing the posterior margins of the eyes; the clypeus has a short medial carina; the antennal scapes surpass the posterolateral corners of the head; the entire mesosoma is smooth and shiny with superficial reticulate sculpturing; the posterior face of the promesonotum is concave before reaching the propodeum; the propodeal spines are very long and slightly curved exceeding the height of the promesonotum; the gaster is uniformly brown, lacking the white translucent spot on the anterior part of the first gastric tergite; and has sparse, short, appressed setae.

The major workers of *P. epaoensis* are very similar to the majors of *P. tanakarensis*. However, the majors of *P. epaoensis* can be distinguished from majors of *P. tanakarensis* and from majors of other *P. sexspinosa* complex species by the following combination of characters: eyes with 5–6 ommatidia along the longest diagonal axis; in dorsal view sculpturing of the pronotum with transverse, rugulose sculpturing and a characteristic, single, medial, longitudinal ridge that at the posterior end of pronotum splits into transverse ridges that extend laterally, whereas the rest of the mesonotal dorsum is covered with a superficial rugoreticulate sculpturing; the propodeal spines are spinate and long, about as long as the petiole; the postpetiole is trapezoidal and its lateral margins are extended as subtriangular projections; the postpetiolar dorsum is covered by rugoreticulate sculpturing; the gaster is a uniform medium-brown that lacks any semi-translucent/white patch.

### Description

Holotype minor worker (Figs. 11A–11C): HW 0.52, HL 0.58, SL 0.50, EL 0.11, WL 0.64, MNH 0.14, PSL 0.24, PTL 0.24, PTH 0.14, PPL 0.10, PPH 0.11, PNW1 0.28, PNW2 0.51, MNW 0.14, PML 0.51, PW1 0.11, PW2 0.34, PTW 0.10, PPW 0.13, MFL 0.55, CI 93, PSLI 45. *Head* in full-face view ovate; lateral margins of head tapering posteriorly behind level of eyes. Posterior margin of head with shallow medial concavity. Frons smooth and shiny with weak, superficial, reticulate sculpturing. Frontal carinae present, barely surpassing posterior margins of eyes. Genae with prominent longitudinal and reticulate ridges. Posterolateral margin of head with rugoreticulate sculpturing contrasting with smooth and shiny medial concavity of the posterior margin. Occipital carina absent. Preoccipital carinae well-developed as low lamellae; posterior corners of carinae forming small teeth that are visible in full-face view. Antennal scapes surpassing posterolateral corner of head by about half the length of first (basal) funicular segment. Antennae with 12 segments, 3-segmented club, and numerous suberect hairs. Antennal scrobes absent. Anterior clypeal margin broadly convex; clypeus smooth with medial carina extending over anterior third of clypeus. Mandibles smooth and shiny with numerous suberect hairs; masticatory margin with large apical and preapical teeth followed by eight teeth of variable size; fourth tooth from apex larger than third. Compound eyes having 7–8 ommatidia along longest axis. Numerous suberect hairs present on lateral and posterior margins of head; hairs almost entirely absent from frons. *Mesosoma* in lateral view with raised, dome-shaped promesonotum with slightly convex dorsal surface; posterior declivity of promesonotal dome excluding the spines concave prior to connecting to propodeum. Pronotal spines long, about as long as the width of the promesonotum, spinate, pointing anterolaterally. Mesonotal spines relatively long, about the size of the petiolar node, subacute, pointing diagonally. Propodeal spines very long, about as long as the petiole, slightly curved, digitiform with sharply pointed apices. Entire mesosoma smooth and shiny with superficial reticulate sculpture and sparse, long, suberect hairs. *Metasoma:* Petiole and postpetiole smooth and shiny but with weak rugulose sculpturing on the lateral and ventral surfaces. Petiole long, more than twice as long as postpetiole, with long anterior peduncle; in lateral view, petiolar node subtriangular; in dorsal view, petiolar node weakly bilobed with rounded corners. In dorsal view, postpetiole ovate with broadly rounded lateral and posterior margins. Gaster smooth and shiny; first gastric tergite entirely brown, without white patches. Head, meso- and metasoma uniformly light brown with lighter colored appendages including spines on mesosoma. Paratype minor workers (*n* = 12): HW 0.51–0.56, HL 0.56–0.60, SL 0.48–0.56, EL 0.09–0.12, WL 0.59–0.65, MNH 0.13–0.16, PSL 0.23–0.29, PTL 0.23–0.25, PTH 0.14–0.15, PPL 0.10–0.11, PPH 0.11–0.14, PNW1 0.28–0.30, PNW2 0.46–0.54, MNW 0.13–0.16, PML 0.50–0.55, PW1 0.09–0.12, PW2 0.31–0.43, PTW 0.09–0.10, PPW 0.13–0.15, MFL 0.51–0.59, CI 89–98, PSLI 42–55.

Paratype major workers (*n* = 6) (Figs. 12A–12C): HW 1.28–1.36, HL 1.32–1.54, SL 0.59–0.69, EL 0.11–0.16, WL 0.93–1.01, MNH 0.15–0.19, PSL 0.24–0.38, PTL 0.30–0.38, PTH 0.23–0.26, PPL 0.16–0.19, PPH 0.13–0.26, PNW1 0.50–0.60, PNW2 0.96–1.10, MNW 0.15–0.31, PML 0.75–0.86, PW1 0.13–0.25, PW2 0.39–0.50, PTW 0.19–0.24, PPW 0.36–0.45, MFL 0.75–0.79, CI 87–97, PSLI 18–28. *Head* in full-face view sub-rectangular; longer than wide (CI 87–97); lateral margins parallel. Posterior margin of head with a deep medial concavity. Frons between frontal carinae and genae longitudinally rugose. Vertex in lateral profile with a shallow transversal impression. Posterolateral corners of head with rugoreticulate sculpture; interspaces smooth and shiny. Ventrolateral margin of head weakly rugoreticulate. Median process of hypostoma absent; hypostomal margin with pairs of inner and outer hypostomal teeth. Frontal lobes present, moderately developed, expanding horizontally, covering antennal insertion into head capsule. Frontal carinae conspicuous, partly overhanging antennal scrobe, extending to approximately 3/5 of head length. Clypeus smooth and shiny, with conspicuous median longitudinal carinae and longitudinally rugose sculpturing around antennal insertions; lateral part of clypeus without any processes. Antennae with 12 segments and 3-segmented club. Compound eyes having 5–6 ommatidia along longest axis. Outer surface of mandibles smooth and shiny; masticatory margin of mandibles with a pair of apical teeth and a single broad basal tooth. Suberect hairs present on posterolateral corners and lateral margins above the eye. *Mesosoma:* In dorsal view, pronotum with transverse, rugulose sculpturing and a single medial, longitudinal ridge that at the posterior end of pronotum splits into transverse ridges that extend laterally; cuticular interspaces smooth and shiny. Mesonotal dorsum with superficial rugo-reticulate sculpturing. Humeral area laterally extended into broad triangular corners. Pronotal spines present, broad at base then significantly constricting at about 2/3 of length with acute apices, pointing anterolaterally. In lateral view, posterior declivity of promesonotal dome with conspicuous prominence extending into mesonotal spines. Mesonotal spines triangular with blunt, tuberculate apices pointing diagonally in lateral view. Metanotal groove shallow, inconspicuous. Propodeal spines spinate and shorter than the petiole. Lateral pronotum weakly reticulate; meso- and metapleuron smooth and shiny. Scattered erect hairs present on lateral margins of mesosomal dorsum. *Metasoma:* Petiole about twice as long as post-petiole. In lateral view, petiolar node subtriangular and bilobed with weak reticulate sculpturing; subpetiolar process absent. Postpetiole trapezoidal with lateral margins produced as acute angular projections. Postpetiolar dorsum with rugoreticulate sculpturing. First gastral tergite appearing smooth and shiny but with fine granular micro-sculpture. Dorsal surface with abundant, long erect to suberect hairs. Head and mesosoma light to medium yellowish-brown and orange-brown; gaster uniformly medium brown, appendages lighter yellowish-brown.

### Etymology

The species epithet *epaoensis* is derived from Epao, which is a village ~23 km NE of Port Vila on the island of Efate where the holotype was collected. The name is formed by adding the Latin adjectival suffix -*ensis*, meaning “from” or “belonging to,” to indicate geographic origin. The epithet is treated as an adjective and agrees in gender with the genus *Pheidole*.

### Type locality

Vanuatu: Efate, 3 km W Epao, 200 m a.s.l., −17.6155, 168.4743, primary forest, stray from sifted leaf litter, leg. C. Rabeling, collection code CR111103-10-09, collection date 03.xi.2011.

### Type material

Holotype minor worker (MCZ-ENT00842361) and paratypes: one minor worker, one major worker (MCZ-ENT00842362) (same collection event as holotype, strays sifted from leaf litter, leg. C. Rabeling, collection code CR111103-10-09, collection date 03.xi.2011); one major worker (same locality as holotype, stray sifted from leaf litter, leg. C. Rabeling, collection code CR111103-06-07, collection date 03.xi.2011); 21 minor workers (same locality as holotype, strays sifted from leaf litter, leg. C. Rabeling, collection code CR111103-07-02, collection date 03.xi.2011); three minor workers (same locality as holotype, strays sifted from leaf litter, leg. C. Rabeling, collection code CR111103-11-04, collection date 03.xi.2011). Holotype and paratypes are deposited in the MCZC. Additional paratypes deposited in the CRC.

### Additional material examined

Vanuatu: nine minors, one major (Efate, 3.5 km NWW Epao, 241 m a.s.l., −17.6127, 168.4686, primary forest, leg. C. Rabeling & E.O. Wilson, collection codes CR111104-02-04, CR111104-06-01, CR111104-08-09, collection date 04.xi.2011); one minor (Efate, Pang Pang, 10 m a.s.l., −17.6749, 168.5450, forest edge, leg. J.K. Wetterer, collection code Wetterer #313); five minors, two majors (Efate, Worowloa (Port Vila), leg. J.K. Wetterer, collection code Wetterer #272).

### Discussion and biology

*Pheidole epaoensis* is known from stray minor and major workers collected from the leaf litter in low elevation coastal and primary forest habitats (10–241 m) on Efate island in Vanuatu. Unfortunately, entire colonies of *P. epaoensis* were not collected and therefore, the biology of this species is unknown. Samples of *P. epaoensis* were included in another study as *Pheidole sexspinosa* (Gray, 2023).

**Figure 13:**
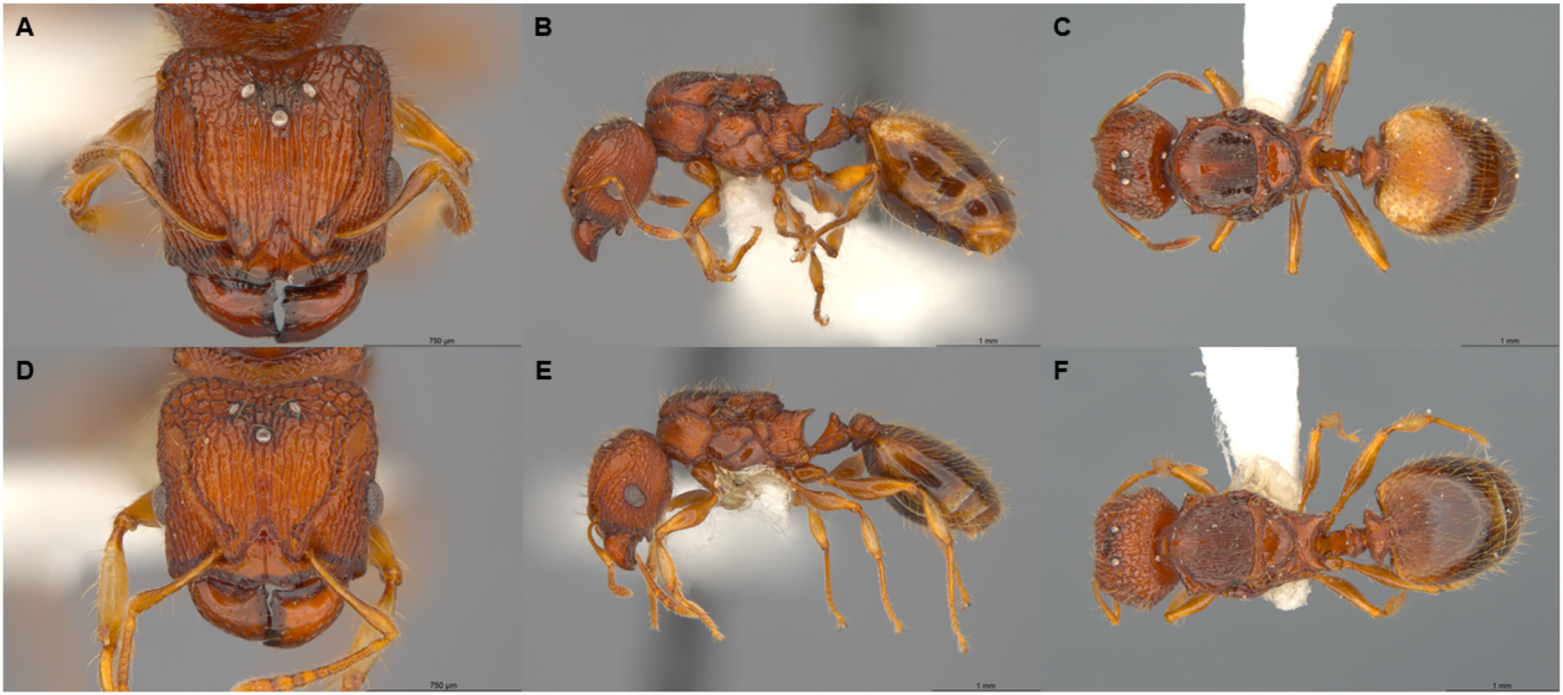
Paratype queens of *Pheidole nivanuatu* (A–C) and *P. tanakarensis* (D–F) in frontal (A, D), lateral (B, E), and dorsal (C, F) views. The scale bars represent 0.75 mm in A & D and 1.0 mm in all other images.

***Pheidole nivanuatu***, Gray, von Adelmannsfelden, Ahmed, Brandstetter, Alvarez, Härtel, Mahmood, Mera-Rodríguez & Rabeling **sp. nov.** (Figs. 11D–11F, 12D–12F, 13A–13C, & 14)

**Figure 14:**
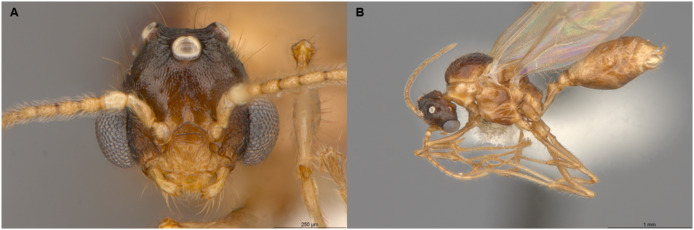
Paratype male of *Pheidole nivanuatu* (A & B) in frontal (A) and lateral (B) views. The scale bars represent 0.25 mm in A and 1.0 mm in B.

[LSID urn:lsid:zoobank.org:act:09AE445D-B034-4CCC-8202-AA6A3055710E]

### Diagnosis

*Pheidole nivanuatu* is a distinct element of the South Pacific ant fauna. It can be differentiated from all other members of the *P. sexspinosa* complex by the following combination of characters: the **minor worker** has an elongated head with parallel lateral margins and the posterior margin has a deep medial concavity; the shape of the mesosoma with its raised promesonotum and flat dorsal surface; the enlarged and rounded mesonotal spines that form a bilobed, shelf-like, posterior extension of the promesonotum; the deeply concave posterior surface of the promesonotal dome; the relatively short antennal scapes that do not reach the posterolateral corners of the head; the short frontal carinae; and the distinct hexagonal shape of the postpetiole. Furthermore, the entire body surface is smooth and shiny, giving it a glass-like appearance. The coloration is also diagnostic with an orange-brown head, metanotum, and propodeum, a reddish-brown promesonotum, yellow-brown coxae and antennal scapes, and the distinctly white and translucent dorsal surfaces of first gastric tergite, pronotal spines, tips of propodeal spines and femora of all legs.

The major worker of *P. nivanuatu* can be recognized by its unique sculpturing of the pronotal dorsum, where the anterior part has transverse, rugulose sculpturing but the posterior half of the pronotum has longitudinal ridges that point towards the midline of the pronotum; the metanotal groove is deep, conspicuous, traversing across the mesosomal dorsum; and the mesonotal dorsum is smooth and shiny. The coloration of the major worker is also unique, similarly to the coloration of the minor worker, with the head and mesosoma dark reddish-brown, mesopleura and propodeum lighter orange-brown, the anterior half of first gastric tergite translucent/white, whereas the rest of the gaster is uniformly medium brown. The appendages are lighter yellowish-brown with light translucent/white patches on femur and tibia.

Superficially, *P. nivanuatu* resembles members of the *Pheidole roosevelti* group (Sarnat, 2008) due to its distinctly smooth and shiny cuticle as well as the long propodeal spines. However, *P. nivanuatu* can be easily differentiated by the presence of the pronotal spines in minor workers.

### Description

Holotype minor worker (Figs. 11D–11F): HW 0.61, HL 0.66, SL 0.58, EL 0.11, WL 0.75, MNH 0.2, PSL 0.38, PTL 0.26, PTH 0.15, PPL 0.13, PPH 0.15, PNW1 0.39, PNW2 0.65, MNW 0.23, PML 0.66, PW1 0.15, PW2 0.54, PTW 0.18, PPW 0.22, MFL 0.60, CI 92, PSLI 62. *Head* in full-face view sub-rectangular; lateral margins of head parallel, not tapering. Posterior margin of head with pronounced medial concavity. Entire surface of head smooth and shiny with few longitudinal rugulae on genae below eyes. Frontal carinae short, not extending beyond posterior margin of eye. Occipital carinae absent. Preoccipital carinae well-developed as conspicuous lamellae; posterior corners of carinae forming small teeth that are visible in full-face view. Antennal scapes not reaching posterolateral corner of head. Antennae with 12 segments, 3-segmented club, and evenly distributed suberect hairs on scape and funiculus. Antennal scrobes absent. Anterior clypeal margin broadly convex but medial section of anterior clypeal margin straight; entire clypeus smooth and shiny, without medial carina. Mandibles smooth and shiny; masticatory margin with large apical and preapical teeth, followed by eight teeth and denticles of variable size; fifth tooth from apex larger than third and fourth. Compound eyes having 5–6 ommatidia along longest axis. Head with scattered, curved, erect, and suberect hairs. *Mesosoma* in lateral view with distinct shape; promesonotum raised to having a flat dorsal surface; mesonotal spines flat, broad, with rounded apices, extending dorsal surface of promesonotal dome posteriorly, forming a broad, bilobed shelf that is overhanging the strongly concave, posterior surface of promesonotal dome. Pronotal spines spinate, sharp, about as long as width of the promesonotum, pointing anterolaterally. Propodeal spines very long, longer than petiole, slightly curved, digitiform with sharply pointed apices. Entire mesosoma smooth and shiny; mesosomal dorsum with scattered, moderately long, erect hairs. *Metasoma:* Petiole and postpetiole smooth and shiny but with weak rugulose sculpturing on the lateral and ventral surfaces. Petiole long, more than twice as long as postpetiole, with long anterior peduncle; in lateral view, petiolar node subtriangular; in dorsal view, distinctly bilobed with lobes extending into tips pointing dorsolaterally. In dorsal view, postpetiole hexagonal with lateral margins triangular with acute tips and broadly rounded posterior margin. Gaster smooth and shiny with scattered suberect hairs. Color distinct and highly variable; head, metanotum and propodeum orange-brown; promesonotum darker, reddish-brown, contrasting with light yellow-brown coxae and antennal scapes; dorsal surface of first gastric tergite, pronotal spines, tips of propodeal spines and femora of all legs distinctly white or translucent. Tibiae, tarsi, funiculi and remainder of gaster medium brown. Paratype minor workers (*n* = 9): HW 0.49–0.63, HL 0.54–0.69, SL 0.49–0.56, EL 0.10–0.13, WL 0.60–0.73, MNH 0.15–0.20, PSL 0.30–0.40, PTL 0.25–0.31, PTH 0.13–0.15, PPL 0.08–0.13, PPH 0.10–0.15, PNW1 0.28–0.41, PNW2 0.48–0.64, MNW 0.16–0.22, PML 0.54–0.66, PW1 0.12–0.16, PW2 0.29–0.60, PTW 0.11–0.17, PPW 0.14–0.22, MFL 0.51–0.61, CI 85–96, PSLI 56–76.

Paratype major workers (*n* = 5) (Figs. 12D–12F): HW 1.16–1.36, HL 1.22–1.44, SL 0.60–0.64, EL 0.11–0.14, WL 0.91–1.01, MNH 0.20–0.21, PSL 0.33–0.39, PTL 0.30–0.38, PTH 0.20–0.25, PPL 0.15–0.19, PPH 0.21–0.25, PNW1 0.51–0.63, PNW2 0.91–1.04, MNW 0.29–0.34, PML 0.81–0.88, PW1 0.14–0.25, PW2 0.53–0.61, PTW 0.20–0.28, PPW 0.39–0.45, MFL 0.74–0.81, CI 93–96, PSLI 27–30. *Head* in full-face view sub-rectangular; slightly longer than wide (CI 93–96); lateral margins parallel. Posterior margin of head with a deep medial concavity. Frons between frontal carinae and genae longitudinally rugose. Vertex in lateral profile with a shallow transversal impression. Posterolateral corners of head with rugoreticulate sculpture; interspaces smooth and shiny. Ventrolateral margin of head longitudinally rugose. Median process of hypostoma absent; hypostomal margin with pairs of inner and outer hypostomal teeth. Frontal lobes present, moderately developed, expanding horizontally, covering antennal insertion into head capsule. Frontal carinae conspicuous, partly overhanging antennal scrobe, extending to approximately 3/5 of head length. Clypeus smooth and shiny, with conspicuous median longitudinal carinae and longitudinally rugose sculpturing around antennal insertions; lateral part of clypeus without any processes. Antennae with 12 segments and 3-segmented club. Compound eyes having 5–6 ommatidia along longest axis. Outer surface of mandibles smooth and shiny; masticatory margin of mandibles with a pair of apical teeth and a single broad basal tooth. Suberect, curved hairs present on posterolateral corners and lateral margins above the eye. *Mesosoma:* In dorsal view, anterior part of pronotum with transverse, rugulose sculpturing; posterior half of pronotum with longitudinal ridges pointing towards the center; cuticular interspaces smooth and shiny. Mesonotal dorsum smooth and shiny. Humeral area evenly rounded. Pronotal spines present with acute apices, broad at base and constricting evenly towards the apices, pointing anterolaterally. In lateral view, posterior declivity of promesonotal dome with conspicuous prominence extending into mesonotal spines. Mesonotal spines triangular with acute apices pointing diagonally in lateral view; in dorsal view, mesonotal spines are connected mesially, extending the mesonotal dorsum posteriorly in a shelf-like projection. Metanotal groove deep, conspicuous, traversing across mesosomal dorsum. Propodeal spines spinate and longer than the petiole. Lateral pronotum with weak, transverse rugae; meso- and metapleuron smooth and shiny. Scattered erect hairs present on lateral margins of mesosomal dorsum. *Metasoma:* Petiole about twice as long as post-petiole. In lateral view, petiolar node subtriangular and bilobed with weak reticulate sculpturing; subpetiolar process absent. Postpetiole trapezoidal with lateral margins produced as acute, short spines. Postpetiolar dorsum smooth and shiny. First gastral tergite appearing smooth and shiny but with fine granular micro-sculpture; color of anterior half distinctly translucent/white. Dorsal surface with abundant, long erect to suberect hairs. Head and mesosoma dark reddish-brown with mesopleura and propodeum lighter orange-brown; anterior half of first gastric tergite translucent/white, rest uniformly medium brown, appendages lighter yellowish-brown with light translucent/white patches on femur and tibia.

Paratype queens (*n* = 2) (Figs. 13A–13C): HW 1.04–1.10, HL 1.06, SL 0.63–0.69, EL 0.26, WL 1.48–1.62, PSL 0.39–0.40, PTL 0.41–0.45, PTH 0.33–0.35, PPL 0.24–0.25, PPH 0.36–0.37, PNW1 0.90–0.92, PNW2 1.00, PW1 0.30–0.32, PW2 0.50–0.51, PTW 0.30–0.34, PPW 0.58–0.59, MFL 0.88, CI 98–104. *Head* in full-face view subrectangular; about as long as wide (CI 98–104); lateral margins parallel. Posterior margin of head concave medially. Presence of three ocelli. Frons and genae longitudinally rugose. Posterolateral corners rugoreticulate; interspaces smooth and shiny. Ventrolateral margin with rugoreticulate sculpturing that weakens towards the posterolateral corners. Median process of hypostoma absent; hypostomal margin with pairs of inner and outer hypostomal teeth. Frontal lobes present, moderately developed, expanding horizontally, covering antennal insertion into head capsule. Frontal carinae extending to approximately 3/4 length of head. Clypeus smooth and shiny, with conspicuous median longitudinal carinae and longitudinally rugose sculpturing around antennal insertions; lateral part of clypeus without any processes. Antennae with 12 segments and 3-segmented club; antennal scrobes with fine rugoreticulate sculpturing. Compound eyes having 15–16 ommatidia along longest axis. Mandibles smooth and shiny; masticatory margin of mandibles with two broad basal teeth then diastema with two broad teeth at the apical margin. *Mesosoma* with usual modifications related to wing-bearing. In dorsal view, pronotum much reduced and with rugoreticluate sculpturing; armed with denticles that point anterolaterally. Mesoscutum with longitudinal, rugulose sculpturing; cuticular interspaces smooth and shiny; smooth and shiny patch present medially on anterior half. Parapsidal line present separating sculptured mesoscutum and lateral strip that is smooth and shiny. Scutoscutellar sulcus deeply trenched and longitudinally furrowed. Mesoscutellar disc smooth and shiny. Anepisternum and katepisternum separated by a shallow mesopleural sulcus. Anepisternum smooth and shiny; katepisternum mostly smooth and shiny except for weak rugose sculpturing along ventral margin. Metapleuron and lateral propodeum with lateral rugose sculpturing. Propodeal spines linear; shorter than petiole. Posterior face of propodeum with weak lateral rugose sculpturing. Suberect hairs present on most mesosomal dorsum. *Metasoma:* Petiole about twice as long as post-petiole. In lateral view, petiolar node subtriangular and bilobed with weak reticulate sculpturing; subpetiolar process absent but with 3–4 denticles along ventral margin; post-petiole with subtriangular lobe along ventral margin. Postpetiole trapezoidal with lateral margins produced as acute angular projections. Postpetiolar dorsum with granular micro-sculpture. First gastral tergite with granular microsculpture like postpetiolar dorsum. Dorsal surface with moderately abundant, long suberect to erect hairs; gaster with additional short, appressed hairs. Head and mesosoma dark reddish-brown; anterolateral corners of first gastric tergite with white/yellowish patch, rest uniformly medium brown; appendages lighter yellowish-brown with light translucent/white patches on femur and tibia.

Paratype males (*n* = 3) (Fig. 14): HW 0.46–0.48, HL 0.50, SL 0.13, EL 0.27, WL 1.14–1.30, PTL 0.31–0.34, PTH 0.16–0.18, PPL 0.15–0.18, PPH 0.18, PNW1 0.78–0.84, PW1 0.27–0.28, PTW 0.16–0.17, PPW 0.24–0.25, MFL 0.81–0.88, CI 0.93–0.95. *Head* in full-face view ovate; slightly longer than wide (CI 0.93–0.95). Posterior margin concave medially. Entire head with weak reticulate sculpturing. Clypeus with weak rugulose sculpturing; anterior margin broadly convex. Mandibles thin with two broad teeth; without diastema. Antennae 13-segmented; basal segment of funiculus ovoid in shape; antennal scrobe absent. Entire posterior margin of head with long suberect hairs. *Mesosoma* with typical modifications related to wing-bearing. In lateral view, dome-shaped and raised. Mesoscutum mostly smooth and shiny but with weak reticulate sculpturing medially. Notaulus distinct with reticulate sculpturing. Parapsidal line weak and indistinct. Scutoscutellar sulcus deeply trenched and longitudinally furrowed. Oblique mesopleural sulcus weakens anteriorly. Anepisternum and katepisternum smooth and shiny. Lower metapleuron smooth and shiny, upper metapleuron weakly rugulose. Propodeum with rugoreticulate sculpturing. *Metasoma:* Petiole about twice as long as post-petiole. Petiole mostly smooth and shiny but with weak transverse sculpturing along the lateral and dorsal margin. Petiolar node subtriangular and bilobed. In dorsal view, post-petiole wider than long. Post-petiole smooth and shiny. Lateral margins of post-petiole broadly convex and rounded with a weak protuberance. Gaster smooth and shiny. Dorsal surface with moderately long, scattered suberect hairs. Head and mesosomal dorsum dark brown; rest of body light brown to yellow.

### Etymology

The species epithet *nivanuatu* is derived from the demonym “Ni-Vanuatu,” referring to the indigenous people of Vanuatu. The name honors the cultural heritage, resilience, and ecological stewardship of the Ni-Vanuatu people. The formation of the name follows Latinization conventions, with *nivanuatu* used as an indeclinable noun in apposition.

### Type locality

Vanuatu: Espiritu Santo, Mount Saratsi, 9 km E Penaoru, 1431 m a.s.l., −14.9716, 166.6901, primary forest, collected from foraging column at night, leg. C. Rabeling, collection code CR120624-14, collection date 24.vi.2012.

### Type material

Holotype minor worker (MCZ-ENT00842363) plus paratypes: 16 minor workers, one alate queen (same collection event as holotype, leg. C. Rabeling, collection code CR120624-14, collection date 24.vi.2012); 200 minor workers, one major worker (MCZ-ENT00842364) and 10 additional major workers, one dealate queen (MCZ-ENT00842365), one male (MCZ-ENT00842366) and 16 additional males (same locality as holotype but 1,311 m a.s.l., −14.9776, 166.6788, leg. C. Rabeling, collection code CR120624-16, collection date 24.vi.2012); one minor worker (same locality as holotype but 1,311 m a.s.l., −14.9776, 166.6788, leg. C. Rabeling, collection code CR120624-13, collection date 24.vi.2012); 162 minor workers, 15 major workers, six males (same locality as holotype but 1,311 m a.s.l., −14.9776, 166.6788, leg. C. Rabeling, collection code CR120624-17, collection date 24.vi.2012). Holotype and paratypes deposited in the MCZC. Additional paratypes deposited in the CRC.

### Additional material examined

Vanuatu: four minors (Espiritu Santo, “Conservation Site,” 5 km Penaoru, 380 m a.s.l., −14.9471, 166.6335, primary forest, leg. C. Rabeling, collection code CR120630-04-06, collection date 30.vi.2012); 10 minors (Espiritu Santo, Mount Tanakar, 4.5 km NW Tatafo, 705 m a.s.l., −15.3682, 166.9780, primary forest, leg. C. Rabeling & E.O. Wilson, collection code CR111114-04-07, collection date 14.xi.2011, collection codes CR111116-02-03, CR111116-04-04, CR111116-12-02, CR111116-13-06, CR111116-19-01, collection date 16.xi.2011); three minors (Espiritu Santo, Mount Voutemele, 11 km SE Penaoru, 825 m a.s.l., −15.0387, 166.6868, primary forest, leg. C. Rabeling, collection codes CR120702-14-02, CR120702-18-10, CR120702-20-12, collection date 02.vii.2012); two minors (Espiritu Santo, Mount Voutemele, 11 km SE Penaoru, 879 m a.s.l., −15.0430, 166.6834, primary forest, leg. C. Rabeling, collection codes CR120702-22-06, CR120702-29-05, collection date 02.vii.2012).

### Discussion and biology

*Pheidole nivanuatu* is so far known only from mid to high elevation primary forest habitats (380–1,431 m) on the island Espiritu Santo in Vanuatu. One colony was collected on Mount Saratsi (type locality) found nesting within a rotting log that consisted of multiple chambers (collection code CR120624-16). One observation of minor workers and a single alate queen in a foraging column at night suggests nocturnal habits. Additional minors, majors, queens, and males were collected from the leaf litter.

A few stray minor workers from Mount Voutemele appear to show an intermediate phenotype with *P. tanakarensis* by having a head that slightly tapers posteriorly and possesses more linear mesonotal spines. However, one minor worker from this population was included in the UCE phylogeny (sample VAN237 in this study) and was nested in the *P. nivanuatu* clade suggesting that these traits are sometimes but rarely variable.

In previous studies, *P. nivanuatu* was referred to as *Pheidole* spVAN05 (Gray, 2023) and as *Pheidole* epem198 (Economo et al., 2015; Sarnat et al., 2017).

***Pheidole tanakarensis*** Gray, von Adelmannsfelden, Ahmed, Brandstetter, Alvarez, Härtel, Mahmood, Mera-Rodríguez & Rabeling **sp. nov.** (Figs. 11G–11I, 12G–12I & 13D–13F)

[LSID urn:lsid:zoobank.org:act:05920595-CD01-4350-AA37-A1A80D4D9FE8]

### Diagnosis

*Pheidole tanakarensis* is so far known to occur in Vanuatu on Espiritu Santo, where it occurs in direct sympatry with *P. nivanuatu*, and on Maewo. *Pheidole tanakarensis* can be differentiated from all other *P. sexspinosa* complex species by the following combination of characters: the minor workers have long frontal carinae extending to the posterior margin of the head; a prominent clypeal carina; relatively short antennal scapes that barely reach the posterolateral corners of the head; the frons of the head, the mesosoma, and the gaster are smooth and covered by a fine granular microsculpture giving a matte appearance; the posterior face of the promesonotal dome is straight, not concave; the propodeal spines are relatively short and straight; the postpetiole is hexagonal in shape with subtriangular lateral margins; and the gaster has a white translucent patch on the anterior portion of the first gastric tergite.

The major worker of *P. tanakarensis* is very similar to the major of *P. epaoensis*. If available, it is best to identify this species by the minor workers. However, the majors of *P. tanakarensis* can be distinguished from majors of *P. epaoensis* and from majors of other *P. sexspinosa* complex species by the following combination of characters: eyes slightly larger with 6–7 ommatidia along the longest diagonal axis; sculpturing of the mesosoma in dorsal view with the anterior part of pronotum with transverse, rugulose sculpturing, whereas the posterior half of pronotum has longitudinal ridges pointing towards the lateral margins of mesosoma; the mesonotal dorsum has weak, longitudinal rugae; the propodeal spines are spinate and shorter than the petiole; the postpetiole is trapezoidal and the lateral margins are extended as acute, short spines; the postpetiolar dorsum is smooth with fine, granular sculpturing; and the color of the gaster with the tergites being medium to dark brown but with two semi-translucent/white patches covering the anterior dorsolateral corners.

### Description

Holotype minor worker (Figs. 11G–11I): HW 0.61, HL 0.66, SL 0.53, EL 0.11, WL 0.75, MNH 0.13, PSL 0.25, PTL 0.30, PTH 0.18, PPL 0.11, PPH 0.14, PNW1 0.34, PNW2 0.63, MNW 0.18, PML 0.61, PW1 0.16, PW2 0.39, PTW 0.13, PPW 0.18, MFL 0.56, CI 92, PSLI 41. *Head* in full-face view ovate; lateral margins of head tapering posteriorly behind level of eyes. Posterior margin of head with shallow medial concavity. Frons smooth but with fine granular microsculpture giving matte appearance; longitudinal rugae present towards lateral margins of frons, gradually transitioning into rugoreticulate sculpturing towards posterior margin of head. Frontal carinae present, extending beyond posterior margins of eyes; carinae discontinuous, running parallel to longitudinal rugae until reaching reticulate sculpture on posterolateral corners of head. Genae with reticulate sculpturing above and below the level of the eyes. Posterolateral corners with pronounced reticulate sculpturing; reticulation weaker at medial concavity of posterior margin. Occipital carina absent. Preoccipital carinae well-developed as low lamellae, posterior corners of carinae forming small teeth visible in full-face view. Antennal scapes barely reaching posterolateral corner of head. Antennae with 12 segments, 3-segmented club with abundant, evenly distributed, appressed hairs and few erect hairs on scape and funiculus. Antennal scrobes absent. Anterior clypeal margin broadly convex; clypeus smooth and shiny with prominent medial carina extending longitudinally over clypeus. Mandibles smooth and shiny with numerous long, suberect hairs; masticatory margin with two large apical teeth followed by six preapical teeth of variable size; fourth tooth from apex larger than third. Compound eyes having 7–8 ommatidia along longest axis. Short, suberect, curved hairs on posterior margin of head; longer erect hairs on frons and ventral side of head. *Mesosoma* in lateral view promesonotum raised to a low dome with convex dorsal surface; posterior declivity of promesonotal dome straight, not concave, prior to connecting to propodeum. Pronotal spines long, about as long as the width of the promesonotum, spinate, pointing anterolaterally. Mesonotal spines short, about half the size of the petiolar node, forming triangular, subacute spines with broad bases, pointing diagonally. Propodeal spines almost straight, spinate, about as long as the petiole, pointing posterolaterally in dorsal view. Mesosomal surface smooth with fine granular microsculpture giving matte appearance; few rugulae present on dorsal face of propodeum. Promesonotal dorsum with few erect hairs. *Metasoma:* Petiole with long anterior peduncle, twice as long as postpetiole; in lateral view, petiolar node subtriangular; in dorsal view, petiolar node weakly bilobed with rounded corners; surface smooth and shiny but with striate sculpturing on the lateral and ventral surfaces, and light rugulose sculpturing on dorsum of petiolar node. Postpetiole smooth and shiny but with sculpturing on ventral surface. In dorsal view, postpetiole hexagonal with subtriangular lateral margins and broadly rounded posterior margin. Gaster smooth with fine granular microsculpture giving matte appearance and few, scattered, appressed hairs. Head and gaster medium to dark brown with anterior third of first gastric tergite distinctly translucent with white patches; mesosoma darker reddish-brown; legs, antennae, tips of mesosomal spines yellowish-brown. Paratype minor workers (*n* = 9): HW 0.46–0.63, HL 0.50–0.69, SL 0.43–0.53, EL 0.09–0.12, WL 0.51–0.71, MNH 0.10–0.13, PSL 0.19–0.25, PTL 0.23–0.26, PTH 0.13–0.18, PPL 0.08–0.11, PPH 0.10–0.15, PNW1 0.23–0.35, PNW2 0.43–0.56, MNW 0.14–0.19, PML 0.44–0.63, PW1 0.10–0.14, PW2 0.29–0.44, PTW 0.08–0.14, PPW 0.11–0.18, MFL 0.41–0.60, CI 90–95, PSLI 37–46.

Paratype major workers (*n* = 7) (Figs. 12G–12I): HW 1.16–1.30, HL 1.26–1.44, SL 0.50–0.63, EL 0.13–0.16, WL 0.91–1.00, MNH 0.13–0.19, PSL 0.28–0.32, PTL 0.31–0.36, PTH 0.19–0.26, PPL 0.13–0.19, PPH 0.19–0.24, PNW1 0.53–0.63, PNW2 0.94–1.08, MNW 0.25–0.31, PML 0.71–0.86, PW1 0.13–0.24, PW2 0.33–0.48, PTW 0.17–0.25, PPW 0.28–0.44, MFL 0.66–0.79, CI 85–97, PSLI 22–26. *Head* in full-face view rectangular; longer than wide (CI 84–97); lateral margins parallel. Posterior margin of head with a deep medial concavity. Frons between frontal carinae and genae longitudinally rugose. Vertex in profile with a shallow transversal impression. Posterolateral corners of head rugo-reticulate sculpture; interspaces smooth and shiny. Ventrolateral margin of head weakly rugo-reticulate. Median process of hypostoma absent; hypostomal margin with pairs of inner and outer hypostomal teeth. Frontal lobes present, moderately developed, expanding horizontally, covering antennal insertion into head capsule. Frontal carinae conspicuous, partly overhanging antennal scrobe, extending to approximately 3/5 of head length. Clypeus smooth and shiny, with conspicuous median longitudinal carinae and longitudinally rugose sculpturing around antennal insertions; lateral part of clypeus without any processes. Antennae with 12 segments and 3-segmented club. Compound eyes having 6–7 ommatidia along longest axis. Outer surface of mandibles smooth and shiny; masticatory margin of mandibles with a pair of apical teeth and a single broad basal tooth. Suberect, curved hairs present on posterolateral corners and lateral margins above the eye. *Mesosoma:* In dorsal view, anterior part of pronotum with transverse, rugulose sculpturing; posterior half of pronotum with longitudinal ridges pointing towards the lateral margins of mesosoma; cuticular interspaces smooth and shiny. Mesonotal dorsum with weaker, longitudinal rugae. Humeral area laterally extended into broad triangular corners. Pronotal spines present, broad at base then significantly constricting at about 2/3 of length with acute apices, pointing anterolaterally. In lateral view, posterior declivity of promesonotal dome with conspicuous prominence extending into mesonotal spines. Mesonotal spines triangular with diffuse, tuberculate apices pointing diagonally in lateral view. Metanotal groove shallow, inconspicuous. Propodeal spines spinate and shorter than petiole. Lateral pronotum weakly reticulate, meso- and metapleuron smooth and shiny. Scattered erect and suberect hairs present on lateral margins of mesosomal dorsum. *Metasoma:* Petiole about twice as long as post-petiole. In lateral view, petiolar node subtriangular and bilobed with weak reticulate sculpturing; subpetiolar process absent. Postpetiole trapezoidal with lateral margins produced as acute, short spines. Postpetiolar dorsum with fine granular sculpturing. First gastral tergite appearing smooth and shiny but with fine granular micro-sculpture; color of gaster medium to dark brown with two semi-translucent/white patched covering the anterior dorsolateral corners. Dorsal surface with abundant, long erect to suberect as well short appressed hairs. Head and mesosoma dark reddish-brown, gaster uniformly medium to dark brown with white patches, appendages lighter yellowish-brown to medium brown.

Paratype queens (*n* = 3) (Figs. 13D–13F): HW 1.04–1.16, HL 1.02–1.18, SL 0.56–0.63, EL 0.23–0.27, WL 1.40–1.48, PSL 0.34–0.37, PTL 0.40–0.50, PTH 0.33–0.34, PPL 0.25–0.28, PPH 0.35–0.38, PNW1 0.75–0.85, PNW2 0.90–1.05, PW1 0.30–0.35, PW2 0.45–0.49, PTW 0.25–0.28, PPW 0.50–0.58, MFL 0.76–0.89, CI 98–102, PSLI 32–33. *Head* in full-face view subrectangular; about as long as wide (CI 98–102); lateral margins parallel. Posterior margin concave medially. Presence of three ocelli. Frons and genae longitudinally rugose. Posterolateral corners rugoreticulate; interspaces smooth and shiny. Ventrolateral margin with rugoreticulate sculpturing that weakens towards the posterolateral corners. Median process of hypostoma absent; hypostomal margin with pairs of inner and outer hypostomal teeth. Frontal lobes present, moderately developed, expanding horizontally, covering antennal insertion into head capsule. Frontal carinae extends to approximately 3/4 length of the head. Clypeus smooth and shiny, with conspicuous median longitudinal carinae and longitudinally rugose sculpturing around antennal insertions; lateral part of clypeus without any processes. Antennae with 12 segments and 3-segmented club; antennal scrobes with fine rugoreticulate sculpturing. Compound eyes having 15–16 ommatidia along longest axis. Mandibles smooth and shiny; masticatory margin of mandibles with two broad basal teeth then diastema with two broad teeth at the apical margin. *Mesosoma* with typical modifications related to wing-bearing. In dorsal view, pronotum much reduced and with rugoreticluate sculpturing; armed with denticles that point anterolaterally. Mesoscutum with longitudinal, rugulose sculpturing throughout; cuticular interspaces smooth and shiny. Parapsidal line weakly present separating sculptured mesoscutum and sculptured lateral strip. Scutoscutellar sulcus moderately trenched and longitudinally furrowed. Mesoscutellar disc mostly smooth and shiny, weak rugulose sculpturing towards posterolateral margins. Anepisternum and katepisternum separated by a shallow mesopleural sulcus. Anepisternum with weak rugulose sculpturing along dorsal margin; katepisternum with rugulose sculpturing on posteroventral half. Metapleuron and lateral propodeum with rugose sculpturing. Propodeal spines linear, shorter than petiole. Posterior face of propodeum with fine rugoreticulate sculpturing. Suberect hairs present on most mesosomal dorsum. *Metasoma:* Petiole about twice the length of post-petiole. In lateral view, petiolar node subtriangular and bilobed with weak reticulate sculpturing; subpetiolar process absent but with two denticles directly under petiolar node; post-petiole with subtriangular lobe along ventral margin. Postpetiole trapezoidal with lateral margins produced as acute angular projections. Postpetiolar dorsum with granular micro-sculpture. First gastral tergite with granular microsculpture like postpetiolar dorsum. Dorsal surface with moderately abundant, long suberect to erect hairs; gaster with additional short, appressed hairs. Head, mesosoma, and gaster dark reddish-brown; appendages lighter yellowish-brown with light translucent/white patches on tibia.

### Etymology

The species epithet *tanakarensis* is derived from Mount Tanakar, a prominent peak in the center of Espiritu Santo in Vanuatu where the holotype was collected. This name recognizes the ecological significance of Mount Tanakar and its role in harboring unique and understudied insect biodiversity within Vanuatu’s montane habitats. The name is formed by adding the Latin adjectival suffix -*ensis*, meaning “from” or “belonging to,” to denote geographic origin. This epithet is treated as an adjective and agrees in gender with the genus *Pheidole*.

### Type locality

Vanuatu: Espiritu Santo, Mount Tanakar, 4.5 km NW Tatafo, 705 m a.s.l., −15.3682, 166.9780, primary forest, colony nesting under bark of rotting branch, leg. C. Rabeling & E.O. Wilson, collection code CR111116-43, collection date 16.xi.2011.

### Type material

Holotype minor worker (MCZ-ENT00842367) plus paratypes: 96 minor workers, one major worker (MCZ-ENT00842368) and 46 additional major workers, one dealate queen (MCZ-ENT00842369) (same collection event as holotype, leg. C. Rabeling & E.O. Wilson, collection code CR111116-43, collection date 16.xi.2011); thee minor workers (same locality as holotype, leg. C. Rabeling & E.O. Wilson, collection code CR111114-29, collection date 14.xi.2011); one minor worker (same locality as holotype, leg. C. Rabeling & E.O. Wilson, collection code CR111114-03-06, collection date 14.xi.2011). Holotype and paratypes deposited in the MCZC. Additional paratypes deposited in the CRC.

### Additional material examined

Vanuatu: two minors (Espiritu Santo, “Butmas,” 19 km NW Luganville, 505 m a.s.l., −15.3489, 167.0026, forest edge, leg. C. Rabeling & E.O. Wilson, collection code CR111114-26, collection date 14.xi.2011); nine minors, eight majors (Espiritu Santo, Oyster Island, 16 km NNE Luganville, 1 m a.s.l., −15.3757, 167.1941, human dominated, leg. C. Rabeling, collection codes CR120613-01-03, CR120613-01-07, CR120613-05-05, CR120613-06, collection date 13.vi.2012); six minors, two majors (Espiritu Santo, “Conservation Site,” 5 km Penaoru, 380 m a.s.l., −14.9471, 166.6335, primary forest, leg. C. Rabeling, collection code CR120630-10-03, collection date 30.vi.2012); one minor, one queen (Espiritu Santo, Mount Saratsi, 9 km E Penaoru, 671 m a.s.l., −14.9861, 166.6564, primary forest, leg. C. Rabeling, collection codes CR120624-32-07, CR120624-37-12, CR120624-37-15, collection date 24.vi.2012); six minors (Espiritu Santo, Mount Saratsi, 9 km E Penaoru 950 m a.s.l., −14.9847, 166.6616, primary forest, leg. C. Rabeling, collection code CR120625-54, collection date 25.vi.2012); two minors (Espiritu Santo, Mount Voutemele, 11 km SE Penaoru, 825 m a.s.l., −15.0387, 166.6868, primary forest, leg. C. Rabeling, collection code CR120702-04-03, collection date 02.vii.2012); two minors (Maewo, “Norovoro”, 2 km N Baitora, village, leg. B. Malkin, collection code Malkin_ 19580829 (assigned by authors), collection date 29.viii.1958).

### Discussion and biology

*Pheidole tanakarensis* occurs in a variety of habitats spanning human dominated low elevation habitats to high elevation primary forest habitats on Espiritu Santo (1–950 m). Additional populations were found on the adjacent Oyster Island and Maewo in Vanuatu. A colony consisting of one queen, 97 minor workers, and 47 major workers was collected from under bark of a rotting branch (holotype collection event). Another colony fragment on Oyster Island consisting of 20 minor workers and nine major workers was collected from rotting roots and the soil below (collection code CR120613-06). Additional queens, minor workers, and major workers were collected from the leaf litter.

In most populations, both minor and major workers possess a semi-translucent white to yellow patch on the first gastral tergite like other widespread forms in the *P. sexspinosa* complex. The only Vanuatuan populations of *P. tanakarensis* that do not possess this trait are from Mount Saratsi and the major workers from Oyster Island. One minor worker from the Mount Saratsi population was included in the UCE phylogeny (VAN221) and is phylogenetically nested within other populations of *P. tanakarensis* suggesting that the coloring of the first gastral tergite is a variable trait.

Minor workers of *P. tanakarensis* resemble other populations of what is currently considered *P. sexspinosa* in terms of color, propodeal spine morphology, and cephalic sculpturing. However, minor workers of *P. tanakarensis* lack standing erect hairs as seen in other populations of *P. sexspinosa*.

In other studies, *P. tanakarensis* was referred to as *Pheidole sexspinosa* (Gray, 2023) and *Pheidole* emsm106 (Economo et al., 2015; Sarnat et al., 2017). In previous phylogenetic studies, as well as in our COI phylogenies (Fig. 8), individuals were inferred as part of the clade that includes Vanuatuan endemics, which is sister to all other *P. sexspinosa* populations that were analyzed. Therefore, we consider *P. tanakarensis* a new species that is presumably endemic to Vanuatu and distinct from other populations belonging to the *P. sexspinosa* complex in the South Pacific region.

## Course survey results

**Figure 15:**
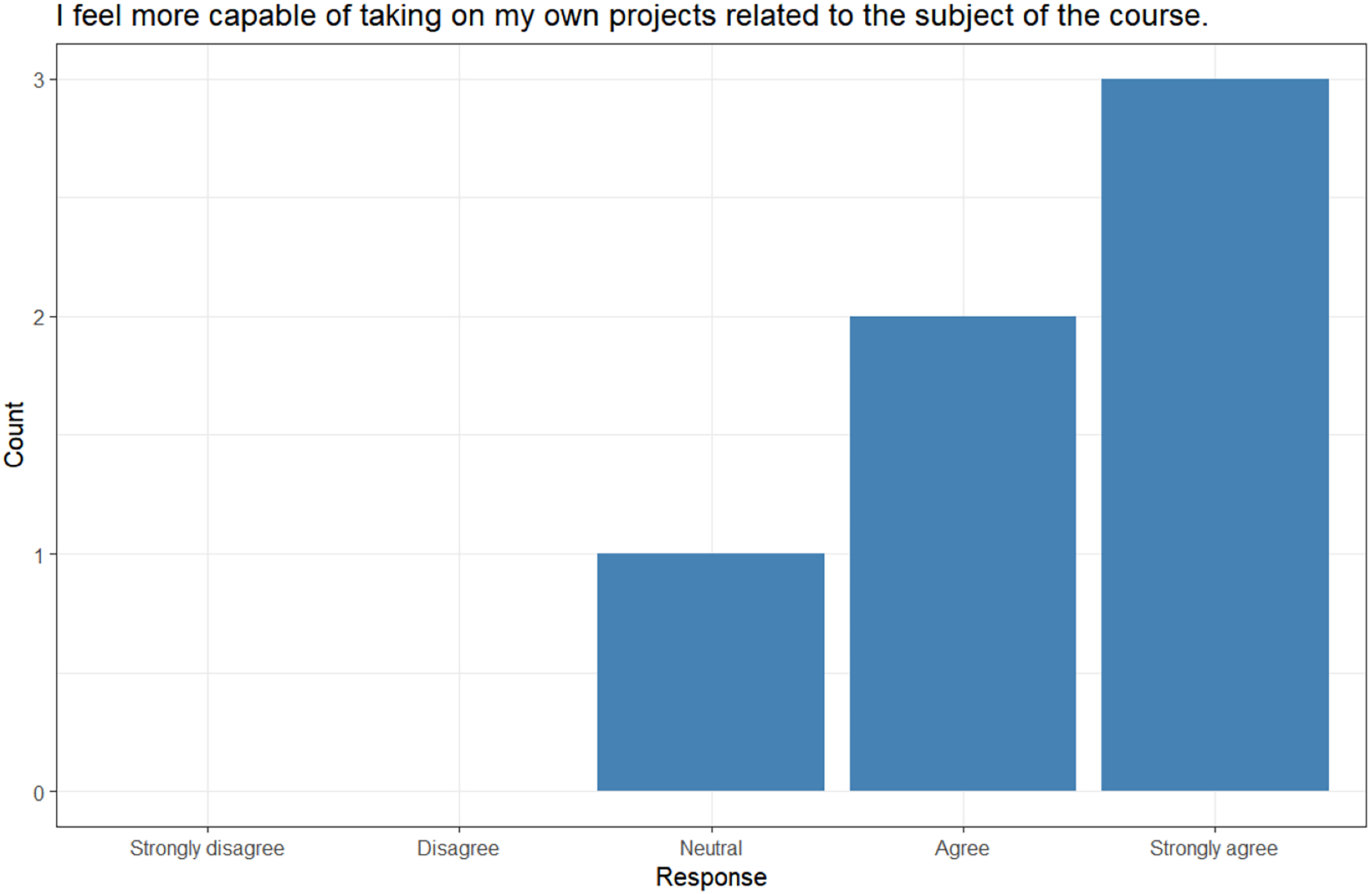
Results from a post-course survey asking student participants to rate their agreement with a statement designed to assess scientific self-efficacy.

After the integrative taxonomy course, students completed an anonymous survey and rated their agreement with the following statement designed to assess scientific self-efficacy: “I feel more capable of taking on my own projects related to the subject of the course.” Responses were strongly skewed toward high self-efficacy: five of six students agreed or strongly agreed with the statement, and no students disagreed (Fig. 15; Supplemental Information 5, Table S1). Students also reported that “It was really interesting to see the whole taxonomic work [process]…from collecting specimens to analyzing the specimens, to making hypotheses and to finally [writing] a paper draft about the findings” and “I have never felt such a strong sense of community in a course before”.

## Discussion

As part of a case-based learning course of integrative taxonomy with a small cohort of university students, we combined quantitative morphometrics, phylogenomics, and biogeography to delimit the *Pheidole sexspinosa* species complex in Vanuatu. The integrative taxonomic results support the presence of three distinct endemic species that we described here as *P. epaoensis*, *P. nivanuatu*, and *P. tanakarensis*. Based on the UCE data and phylogenetic analyses, each of the three Vanuatuan species are reciprocally monophyletic and do not show evidence of gene flow, including populations living in direct sympatry such as *P. tanakarensis* and *P. nivanuatu* on Espiritu Santo (Fig. 7; Table 1). The COI phylogenetic analyses, which includes a broader taxon sampling of the *P. sexspinosa* complex from across the Pacific region, recovered the Vanuatuan *P. sexspinosa* complex species as monophyletic within the broader complex but with moderate support (Fig. 8; UFB = 79/BS = 94). A previous phylogeographic study of *Pheidole* ants (Economo et al., 2015) similarly inferred the Vanuatuan species as sister to other *P. sexspinosa* populations, consistent with the COI phylogenies in this study (Fig. 2). These findings support the hypothesis that the Vanuatuan species descended from a common ancestor and have since diversified *in situ* within the archipelago. Based on comparative morphology and quantitative morphometrics, minor workers of the Vanuatuan *P. sexspinosa* complex are readily distinct whereas major workers are less differentiated (Figs. 5 & 6), which suggests that a close analysis of minor workers will likely be important to disentangle species boundaries for the broader *P. sexspinosa* complex. Overall, these results increase the alpha diversity of the *Pheidole sexspinosa* species complex from three to six species and subspecies as well as suggest a need for a revision of the *P. sexspinosa* complex using an integrative framework.

Our study shows there to be three distinct species of the *P. sexspinosa* complex in Vanuatu that are differentiated by morphological characters observed in other regional populations such as differences in facial sculpturing and spine length, especially in the minor workers. Furthermore, a previous phylogeographic study of *Pheidole* ants inferred there to be at least two distinct subclades within the *P. sexspinosa* complex reflecting the presence of different evolutionary lineages (Economo et al., 2015). Given this, we predict that an integrative revision of the broader *P. sexspinosa* complex will reveal a suite of distinct species. In addition, there remain important questions about the phylogenetic placement of the type population of *P. sexspinosa*, which was collected from the remote Polynesian archipelago of Tuvalu some 1,400 km northeast of Vanuatu (Fig. 1). We suspect that the type population of *P. sexspinosa* belongs to the “eastern clade” and is more closely related to the Vanuatuan populations rather than the “western clade” populations in southeast Asia and Micronesia some 4,000 km west of Tuvalu. Thus, what is currently considered as “*P. sexspinosa*” in southeast Asia, *i.e*., in Singapore (Wang, Yamada & Eguchi, 2018), and in Micronesia, *i.e*., in Palau (Economo et al., 2015), are possibly distinct from the *P. sexspinosa* described from Tuvalu. The only molecular phylogeny so far to include populations across a significant range of the *P. sexspinosa* complex was the COI phylogeny from this study (Fig. 8), but it does not provide robust insight into the evolutionary history of the group, nor does it include the type population of *P. sexspinosa* from Tuvalu. Regarding the presence of *P. sexspinosa* in Vanuatu, that remains an open question until a regional taxonomic revision of the complex is carried out and new type specimens are assigned. For now, we refer to the populations in Palau and Singapore as *P. sexspinosa* like in recent studies of the complex and this form is so far not known to be present in Vanuatu. Future integrative taxonomic studies will need to improve the taxon sampling for the *P. sexspinosa* complex to gain better insight into the placement of the type population from Tuvalu as well as the species boundaries and broad evolutionary patterns of the group.

We are amid a global biodiversity crisis that requires conservation efforts worldwide, especially for fragile ecosystems such as tropical Pacific islands like Vanuatu. To facilitate conservation efforts, it is paramount to have robust taxonomic frameworks to identify conservation priorities. Therefore, it is crucial to facilitate the growth of taxonomic skills for young scientists, especially skills in applying an integrative taxonomic framework that combines traditional morphological approaches with molecular and bioinformatic techniques to test biogeographic and evolutionary hypotheses. A collaborative, case-based learning approach of integrative taxonomy is a feasible way to directly transfer these skills to the next generation of biodiversity scientists and taxonomists in an authentic way. Based on student surveys during this study, this collaborative and case-based learning approach to integrative taxonomy gave them a deeper understanding and appreciation for the mechanisms generating biodiversity, provided a sense of real community, and enhanced their scientific self-efficacy by increasing their confidence in taking on their own taxonomic projects (Fig. 15; Supplemental Information 5, Table S1). This result aligns with a recent study that also applied a case-based learning approach for taxonomic training using ants (Williams et al., 2025) as well as other cooperative learning approaches (Johnson, Johnson & Smith, 2014). Therefore, the time is ripe for case-based learning of integrative taxonomy, especially due to the presence of specimen repositories at most institutions as well as the copious amount of molecular data on publicly accessible repositories or part of ongoing studies. We hope that this study on case-based learning of integrative taxonomy inspires other teachers and researchers to involve their students in taxonomy, biodiversity science, and conservation.

## Conclusions

As part of an in-class, case-based approach to teaching integrative taxonomy in higher education, we provide evidence for three newly discovered ant species in the *Pheidole sexspinosa* complex that diversified *in situ* within the Vanuatuan archipelago. Our study also shows a need and provides a foundation for a broader revision of ants in the *P. sexspinosa* complex using an integrative framework. Finally, we advocate that a case-based learning approach to integrative taxonomy is useful for transferring skills in taxonomy, biodiversity science, and conservation to students with little to no previous experience in these fields.

## Supplemental Information

10.7717/peerj.21333/supp-1 Supplemental Information 1 Occurrence records for the Vanuatuan *Pheidole sexspinosa* complex.

10.7717/peerj.21333/supp-2 Supplemental Information 2 Morphological measurements for the Vanuatuan *Pheidole sexspinosa* complex. All measurements are in micrometers.

10.7717/peerj.21333/supp-3 Supplemental Information 3 Information about DNA extracted specimens of Vanuatuan *Pheidole sexspinosa* complex.

10.7717/peerj.21333/supp-4 Supplemental Information 4 Additional morphometric figures of the Vanuatuan *Pheidole sexspinosa* complex.

10.7717/peerj.21333/supp-5 Supplemental Information 5 Course survey and comments from students.

## Additional Information and Declarations

### Field Study Permissions

The following information was supplied relating to field study approvals (*i.e*., approving body and any reference numbers):

Field experiments were approved by the Museum of Comparative Zoology at Harvard University and the government of Vanuatu.

### Data Availability

The following information was supplied regarding data availability:

The species occurrence records used in this study; the raw morphological measurements; locality and collection information for the UCE samples and additional morphometric figures are available in the Supplemental Files.

The R code used for the morphometrics, as well as the raw morphological measurements, are available at Zenodo: Gray, K. W. (2026). A case-based course teaching integrative taxonomy delimits three new ant species in the *Pheidole sexspinosa* complex (Hymenoptera, Formicidae) from Vanuatu [Data set]. Zenodo. https://doi.org/10.5281/zenodo.18070299.

The raw reads generated for this study are available at NCBI Sequence Read Archive (SRA) BioProject: PRJNA1394312.

The extracted COI sequences from UCEs as part of this study are available at GenBank: PX776474–PX776484.

### New Species Registration

The following information was supplied regarding the registration of a newly described species:

Publication LSID: urn:lsid:zoobank.org:pub:BAFC74A4-2BC1-451E-98CD-2B888B28C6DB

Pheidole epaoensis Gray, von Adelmannsfelden, Ahmed, Brandstetter, Alvarez, Härtel, Mahmood, Mera-Rodríguez & Rabeling: urn:lsid:zoobank.org:act:3FF8A4DE-A18B-4671-9E4C-1ABDBC0E1659

Pheidole nivanuatu Gray, von Adelmannsfelden, Ahmed, Brandstetter, Alvarez, Härtel, Mahmood, Mera-Rodríguez & Rabeling: urn:lsid:zoobank.org:act:09AE445D-B034-4CCC-8202-AA6A3055710E

Pheidole tanakarensis Gray, von Adelmannsfelden, Ahmed, Brandstetter, Alvarez, Härtel, Mahmood, Mera-Rodríguez & Rabeling: urn:lsid:zoobank.org:act:05920595-CD01-4350-AA37-A1A80D4D9FE8.
