# Supplementary material for: A case-based course teaching integrative taxonomy delimits three new ant species in the Pheidole sexspinosa complex (Hymenoptera, Formicidae) from Vanuatu
Source: PeerJ. 2026 Jun 18;14:e21333. doi: 10.7717/peerj.21333 (PMC13283368; doi:10.7717/peerj.21333)
Supplement: Supplemental Information 4 [file peerj-14-21333-s004.docx]

**Supplementary morphological analyses of the Vanuatuan *Pheidole sexspinosa* complex**

for project titled

“A case-based course teaching integrative taxonomy delimits three new ant species in the *Pheidole sexspinosa* complex (Hymenoptera, Formicidae) from Vanuatu”


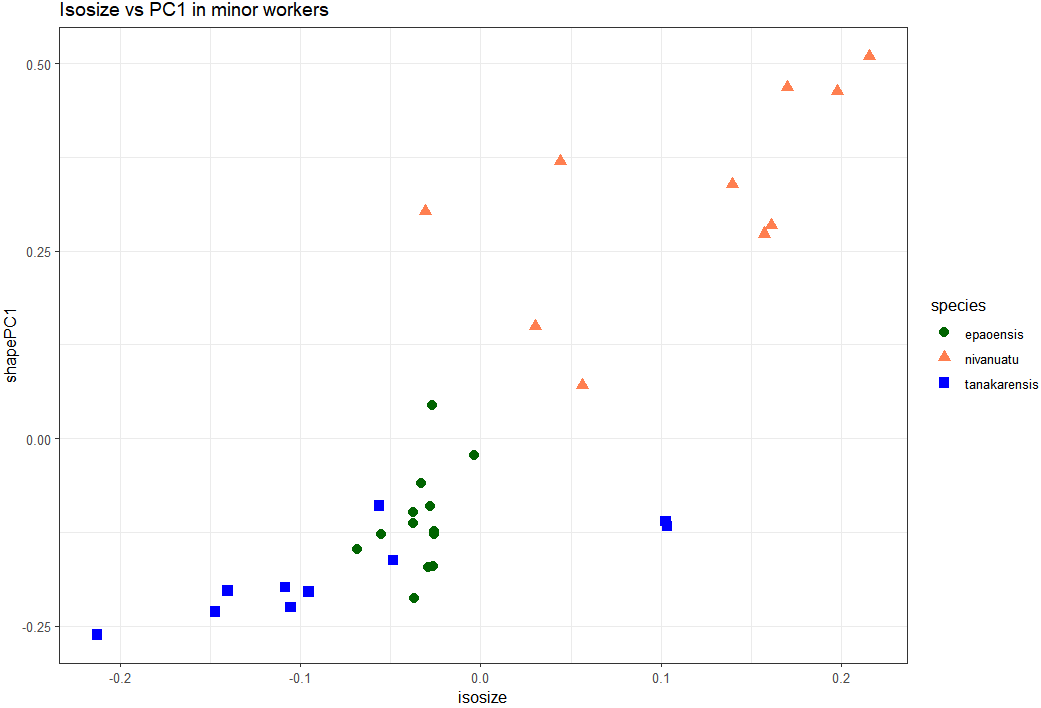


Fig. S1: isosize versus shape PC1 in the minor workers

**
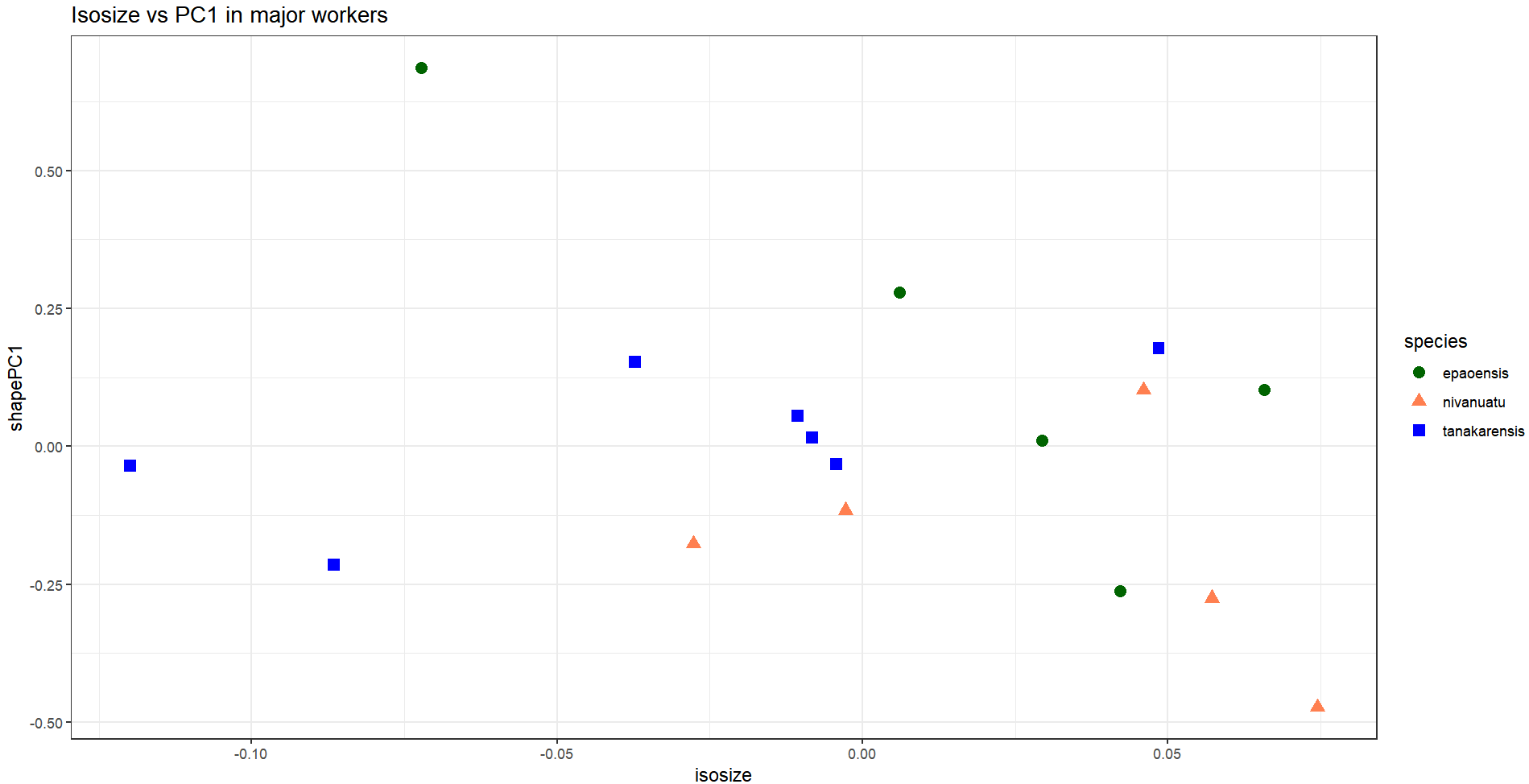
**

Fig. S2: isosize versus shape PC1 in the major workers

**
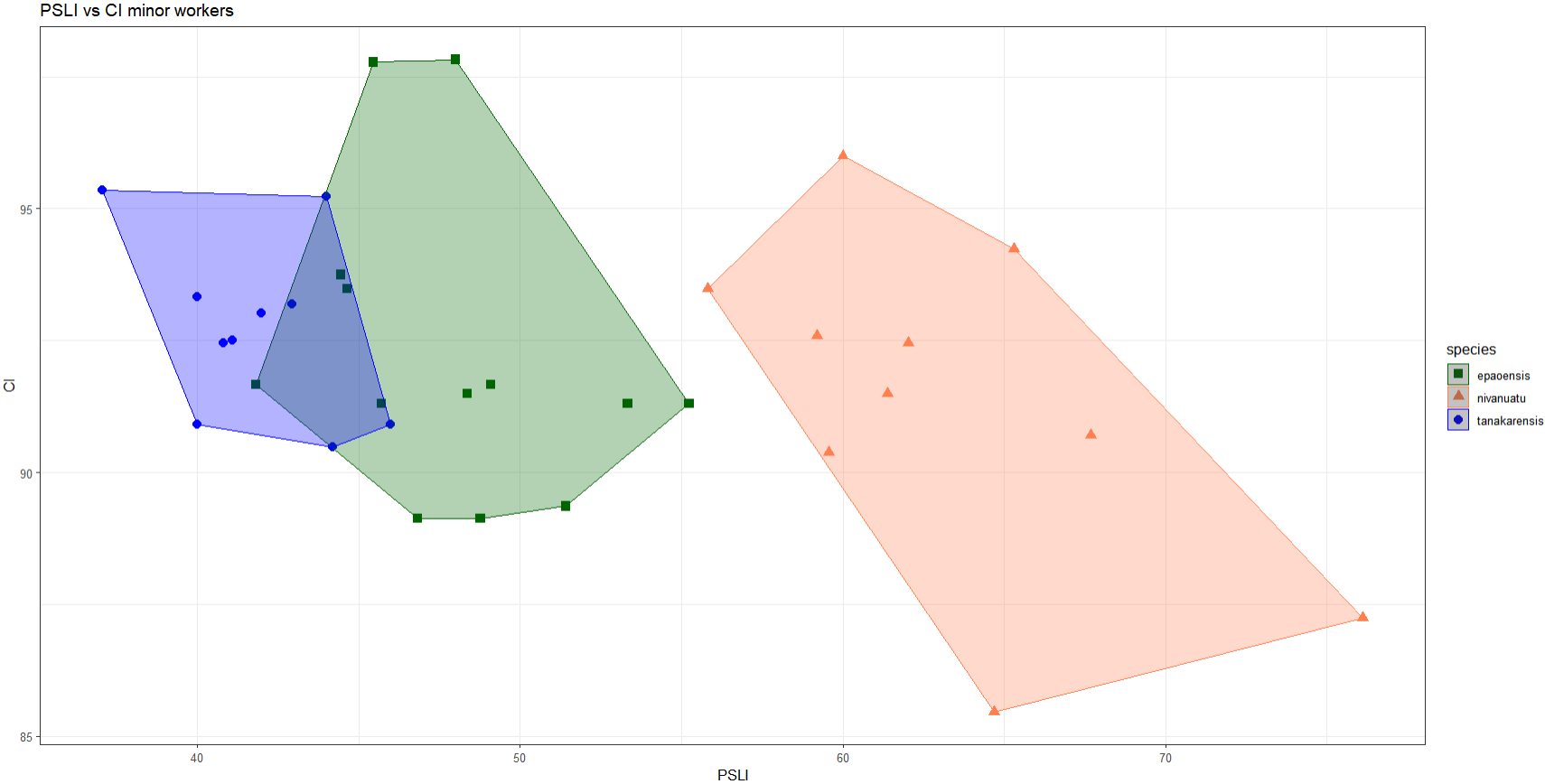
**

Fig. S3: PSLI vs CI in the minor workers

**
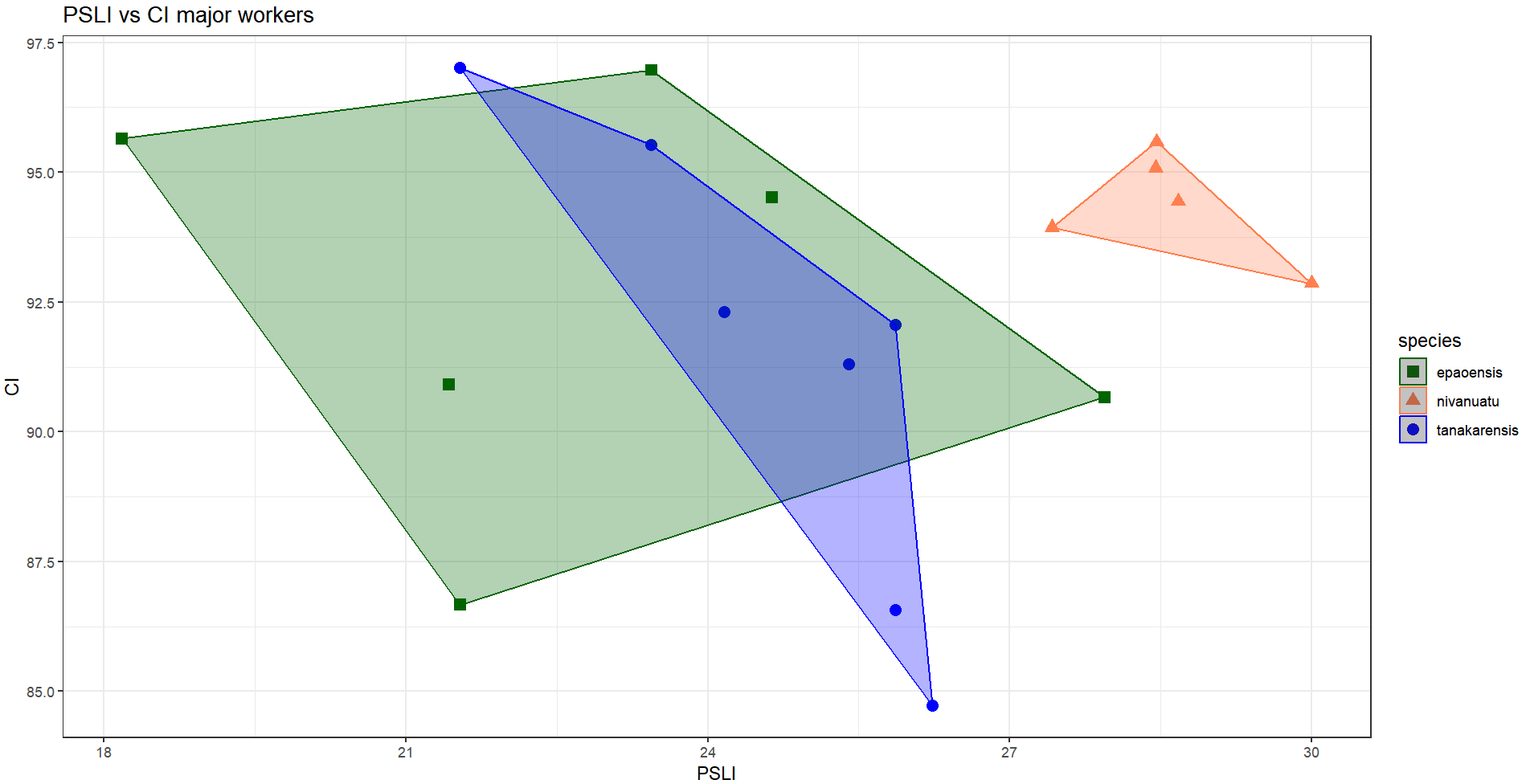
**

Fig. S4: PSLI vs CI in the major workers
